# Generation of Differentiating and Long-Living Intestinal Organoids Reflecting the Cellular Diversity of Canine Intestine

**DOI:** 10.3390/cells9040822

**Published:** 2020-03-28

**Authors:** Nina Kramer, Barbara Pratscher, Andre M. C. Meneses, Waltraud Tschulenk, Ingrid Walter, Alexander Swoboda, Hedwig S. Kruitwagen, Kerstin Schneeberger, Louis C. Penning, Bart Spee, Matthias Kieslinger, Sabine Brandt, Iwan A. Burgener

**Affiliations:** 1 Division of Small Animal Internal Medicine, Department for Small Animals and Horses, University of Veterinary Medicine, 1210 Vienna, Austria; 2 Department of Clinical Sciences, Faculty of Veterinary Medicine, Utrecht University, 3584 Utrecht, The Netherlands; 3 Institute of Pathology, Department for Pathobiology, University of Veterinary Medicine, 1210 Vienna, Austria; 4 Research Group Oncology, Equine Surgery, Department of Small Animals and Horses, University of Veterinary Medicine, 1210 Vienna, Austria

**Keywords:** intestinal organoids, canine intestine, differentiation, organoid culture

## Abstract

Functional intestinal disorders constitute major, potentially lethal health problems in humans. Consequently, research focuses on elucidating the underlying pathobiological mechanisms and establishing therapeutic strategies. In this context, intestinal organoids have emerged as a potent in vitro model as they faithfully recapitulate the structure and function of the intestinal segment they represent. Interestingly, human-like intestinal diseases also affect dogs, making canine intestinal organoids a promising tool for canine and comparative research. Therefore, we generated organoids from canine duodenum, jejunum and colon, and focused on simultaneous long-term expansion and cell differentiation to maximize applicability. Following their establishment, canine intestinal organoids were grown under various culture conditions and then analyzed with respect to cell viability/apoptosis and multi-lineage differentiation by transcription profiling, proliferation assay, cell staining, and transmission electron microscopy. Standard expansion medium supported long-term expansion of organoids irrespective of their origin, but inhibited cell differentiation. Conversely, transfer of organoids to differentiation medium promoted goblet cell and enteroendocrine cell development, but simultaneously induced apoptosis. Unimpeded stem cell renewal and concurrent differentiation was achieved by culturing organoids in the presence of tyrosine kinase ligands. Our findings unambiguously highlight the characteristic cellular diversity of canine duodenum, jejunum and colon as fundamental prerequisite for accurate in vitro modelling.

## 1. Introduction

Gastrointestinal (GI) disorders such as inflammatory bowel disease (IBD), infection-induced GI disorders and cancer have a major negative impact on human health and impose a high financial burden on healthcare systems. Infectious diarrhea is the second leading cause of death among young children [1] and GI cancers are the third most common cancer type worldwide, with one million newly diagnosed cases per year [2]. Potentially lethal GI diseases also affect livestock and companion animals, with enterotoxic bacteria and enteropathogenic viruses being frequently involved in disease onset and progression [3]. Although considerable efforts are made to establish new therapies for human and veterinary GI diseases, mortality rates remain high, because translation of biomedical research into clinical practice is hampered by the lack of epithelial models that reliably mimic the organ and recapitulate disease in patients [4,5,6].

Early attempts to bring the intestinal epithelium to in vitro culture were mostly short-lived, as exemplified by explanted biopsies and epithelial cells, which disintegrated after 72 h and two weeks, respectively [7,8,9]. To establish culture systems for long-lived homogenous cell populations tissue explants were either cultivated in collagen gels or on 3T3 feeder layers on an air-liquid interface [10,11]. However, growth factor-providing (myo)fibroblasts, which are prerequisites for prolonged cultivation in these systems, made them susceptible to inconsistencies from one experiment to another. A major step to overcome these limitations was the establishment of three-dimensional (3D) murine intestinal organoids derived from adult intestinal stem cells [12]. These organoids contained long-lived stem cells that differentiated into the main cell types of murine small intestine such as Paneth cells, enterocytes, enteroendocrine cells and goblet cells. Establishment of this innovative in vitro model was achieved by using a laminin-rich extracellular matrix (Matrigel) and growth media supplemented with epithelial growth factor (EGF), Noggin, and R-spondin as reviewed in detail by Date and Sato [13]. For the cultivation of human intestinal organoids, growth medium had to be supplemented with Wnt3a, gastrin, p38-MAPK inhibitor, nicotinamide and ALK4/5/7 inhibitor, thereby preventing the differentiation into goblet and enteroendocrine cells and retaining enterocytes in a premature state [14]. Withdrawing nicotinamide and p38-MAPK inhibitor induced de novo differentiation, thus shortening the lifespan of the intestinal stem cell pool and rendering this system into an endpoint assay. Further improvement of human 3D intestinal organoid models was achieved by microscaffolds that mimic the size and distance between crypts in transwell assays [15]. Differentiation was promoted by using a growth factor gradient system based on (i) intestinal stem cell-supporting expansion medium in lower wells, and (ii) addition of differentiation medium to upper wells, thereby inducing differentiation of migrating cells to goblet cells, enteroendocrine cells and enterocytes along the modeled crypt/villus axis. However, this technique is unsuitable for, e.g., high-throughput screenings because it is very labor-intensive, time-consuming and hampers down-stream analysis. An approach characterizing receptor tyrosine kinase signaling between the crypt base and its niche managed to omit nicotinamide and p38-MAPK inhibitor usage by a combination of two ligands, i.e., insulin-like growth factor 1 (IGF1) and basic fibroblast growth factor (FGF2). This combination sustains stem cell growth and allows simultaneous differentiation into enteroendocrine and goblet cells, mimicking corresponding natural epithelia more authentically with minimal effort.

In recent years, organoid technology has also been introduced into veterinary research, albeit to a lesser extent [16,17,18,19,20,21,22]. Animal organoid systems that are already available today mainly consist of stem and undifferentiated cells requiring tailor-made species-specific media to allow for differentiation [17]. Interestingly, dogs and their owners share similar environments, food and carcinogenic load, and develop similar GI diseases including GI cancer, infectious disease and IBD [23]. Consequently, canine patients represent an interesting natural model for human GI disorders, even more so since dogs show reduced genetic variation within the majority of breeds [24]. To exploit the full potential of canine intestinal organoids to mimic the corresponding organ accurately, a balance between self-renewal and differentiation of stem cells must be achieved to design a physiologically relevant system. 

Herein, we report the establishment of a novel culture system for canine intestinal organoids that is based on recent findings by Fujii et al. [25] and reliably supports the sustained proliferation of duodenum-, jejunum- and colon-derived stem cells, while concomitantly allowing their differentiation into secretory lineage cells. 

## 2. Materials and Methods

### 2.1. Isolation and Cultivation of Canine Intestinal Organoids

Duodenal, jejunal and colonic samples were obtained from three dogs (two males and one female) that were euthanized due to non-intestinal disease. In addition, duodenal biopsies were taken from one dog undergoing routine gastroduodenal endoscopy. Tissue sampling was approved by the institutional ethics committee in accordance with Good Scientific Practice guidelines and Austrian legislation. Based on the guidelines of the institutional ethics committee, use of tissue material collected during therapeutic excision or post mortem is included in the University’s “owner’s consent for treatment”, which was signed by all patient owners. Intestinal crypts were isolated from tissue samples and biopsies according to established protocols [12]. In short, tissue was incubated with 5 mM EDTA (Sigma-Aldrich, St. Louis, MO, USA) in order to dissociate crypts for 30 min to one hour depending on bowel segment, followed by vigorous shaking until crypts were released. After two washing steps with PBS and Advanced Dulbecco’s modified Eagle’s medium/F12 (DMEM/F12, Invitrogen, Thermo Fisher Scientific, Waltham, MA, USA) 500 crypts were resuspended in 50 µL Matrigel (BD Biosciences, Franklin Lakes, NJ, USA) and seeded per well of a 24-well plate. Following Matrigel polymerization expansion medium was added. Canine intestinal growth media were prepared as indicated in Table 1. Penicillin/streptomycin, HEPES, Glutamax, B27 (with Vitamin A) and N2 supplement were obtained from Invitrogen, Thermo Fisher Scientific. N-acetylcysteine, gastrin and nicotinamide were purchased from Sigma-Aldrich. EGF was provided by Thermo Fisher Scientific. ALK5 kinase inhibitor (A83-01) was obtained from Tocris Bioscience, Bristol, UK. p38 MAPK inhibitor (SB202190) was purchased from Selleck Chemicals, Houston, TX, USA. Human hepatocyte growth factor (HGF), human Noggin, human IGF1 and human FGF2 were provided by PeproTech, Rocky Hill, NJ, USA. Cultrex R-spondin1 cells were obtained from Trevigen, Gaithersburg, MD, USA. Expansion medium was supplemented with 10 µM Rock inhibitor (Y-27632, Selleck Chemicals) for the first two days after isolation, then medium was changed to not-supplemented expansion medium. For isolations using refined medium, EGF and 10 µM Y-27632 were additionally added only for the first two days. Growth medium was changed every two to three days. For weekly passaging at 1:4 to 1:8 split ratios, organoids were harvested, mechanically disrupted using a flame-polished Pasteur pipette. Depending on the splitting ratio, the corresponding quantity of organoid fragments were then embedded in 50 µL fresh Matrigel, seeded per well of a 24-well plate and cultured with either expansion or refined growth medium. To induce differentiation, organoids cultivated in expansion medium were transferred to differentiation medium and analyzed after 4 days. For assessing derivation efficiency in expansion and refined medium, organoids were counted 10 days after initial isolation and projected area was calculated using ImageJ64 (NIH). 

### 2.2. Gene Expression Analysis

Total RNA was purified from duodenal, jejunal and colonic organoids in expansion, differentiation and refined medium and from corresponding intestinal epithelium that was harvested during crypt isolation using ReliaPrep™ RNA Tissue Miniprep System (Promega, Madison, WI, USA) according to instructions of the manufacturer. Individual RNA concentrations were determined spectrophotometrically (Nanodrop One C, Thermo Fisher Scientific) and RNA integrity was checked using Agilent Tape Station 4200 (Agilent, Santa Clara, CA, USA). 500 ng RNA was reverse-transcribed to cDNA with oligo-dT and random hexamer primers according to recommendations of the manufacturer (GoScript™ Reverse Transcription System, Promega). Subsequently, qPCR reactions were carried out using GoTaq^®^ qPCR Master Mix (Promega). Primer sequences are given in Table S1. Amplification conditions were as follows: 2 min of initial denaturation at 95 °C, 40 cycles of 15 s of denaturation at 95 °C, 60 s of annealing/extension at 60 °C and a read step, followed by 10 s of dissociation at 95 °C and a melting curve from 65 °C to 95 °C in 5 s per 0.5 °C increments. Data analysis was performed according to Pfaffl et al. [26] taking PCR efficiency into account. Relative quantification was achieved by normalization of values to the stably expressed canine housekeeping gene hypoxanthine phosphoribosyl transferase (HPRT). Heat maps were generated using Excel conditional formatting of log2 fold changes.

### 2.3. Periodic Acid-Schiff Reaction of Organoid and Tissue Sections

For the generation of formalin-fixed paraffin-embedded (FFPE) samples, organoids were fixed using 2% *v/v* paraformaldehyde (PFA, Merck, Darmstadt, Germany) for 15 min at room temperature, cast in 1.5% *w/v* agarose and dehydrated prior to embedding in paraffin (Sigma-Aldrich). 2.5 µm FFPE sections of organoids derived from duodenum, jejunum and colon and corresponding tissue were stained using PAS-Reaction staining kit and counterstained with Haematoxylin acidic after Mayer according to manufacturer’s instructions (Morphisto, Frankfurt am Main, Germany). Images were acquired with a DMi8 microscope and LASX software (Leica, Wetzlar, Germany). 

### 2.4. Transmission Electron Microscopy of Organoids

Samples were fixed in 3% *v/v* buffered glutaraldehyde (pH 7.4, Merck, Darmstadt, Germany), pre-embedded in 1.5% *w/v* agarose (Invitrogen, Thermo Fisher Scientific), washed three times in 0.1 M phosphate buffer (Soerensen, pH 7.4) afterwards and post-fixed in 1% *v/v* osmium tetroxide (Electron Microscopy Sciences, Hatfield, USA) for 2 h at room temperature. Dehydration was performed in a series of graded ethanol solutions (70%, 80%, 96% and 100%), subsequently infiltrated with propylene oxide (Sigma-Aldrich), followed by increasing ratios of epoxy resin-propylene oxide and finally pure resin (Serva, Mannheim, Germany). After an additional change, the resin was polymerized at 60 °C for 48 h. Semi-thin sections were cut at 0.8 μm and stained with toluidine blue, ultra-thin sections were cut at 70 nm, mounted on copper grids (Gröpl, Tulln, Austria) and stained with uranyl acetate (Fluka Chemie AG, Buchs, CH) and lead citrate (Merck, Darmstadt, Germany). Transmission electron micrographs were made with an EM900 (Zeiss, Oberkochen, Germany).

### 2.5. Proliferation Assay

In order to assess cell proliferation, the Click-iT^®^ EdU Imaging Kit (Invitrogen, Thermo Fisher Scientific) was used. Canine intestinal organoids cultivated in expansion and refined medium were incubated with 5-ethynyl-2’deoxyuridine (EdU) at a final concentration of 10 µM for one hour at 37 °C and were then fixed with 2% *v/v* PFA for 15 min at room temperature. Staining procedure was carried out according to manufacturer’s instructions. DNA was counterstained using 4′,6-Diamidine-2′-phenylindole dihydrochloride (DAPI, Sigma-Aldrich). Confocal images were taken using an LSM 880 (Zeiss). For 3D reconstruction of z-stack images, the Arivis 3D plugin of Zen 2.3 lite (Zeiss) was used.

### 2.6. Viability and Apoptosis Assays

Viability and apoptosis of organoids in the three media compositions were assessed using the RealTime-Glo MT Cell Viability Assay (Promega) and RealTime-Glo Annexin V Apoptosis Assay (Promega). Organoids were trypsinized for 2 min and seeded in 24-well plates with expansion or refined medium, both supplemented with Y-27632 inhibitor. After 2 days in culture, organoids were harvested, counted and 50 organoids were seeded in six wells of white 96-well plate for each condition and assay. Detection reagents were prepared according to manufacturer and added to the different culture media before overlaying the organoids with either expansion, differentiation or refined medium. Luminescence was measured 0 h, 24 h, 48 h and 72 h after adding the substrates using a GloMax plate reader (Promega). Six wells without organoids, but with medium, served as background controls. 

### 2.7. Statistical Evaluation

Data were analyzed and plotted in Prism5 (GraphPad, San Diego, CA, USA). Using a t-test for independent samples (two tailed, unpaired) *p*-values ≤ 0.05 were considered statistically significant. qPCR data were analyzed by two-way ANOVA (factors are growth medium and individual dogs) and Tukey multiple comparison test using R (version 3.3.1). Statistical results are shown in Tables S2–S7. *** *p* < 0.001 ** *p* < 0.01 * *p* < 0.05. *p* < 0.1.

## 3. Results

### 3.1. Expansion Medium Does Not Support Expression of Secretory Lineage Differentiation Markers

Canine intestinal organoids derived from duodenal, jejunal and colonic tissue were generated and cultivated in expansion medium. Initial cell isolates representing the intestinal epithelium after separation from the underlying tissue, as well as low- (≤5) and high-passage (≥10) organoids derived therefrom, were quantitatively assessed for expression of selected cell markers using RT-qPCR. 

**Figure 1:**
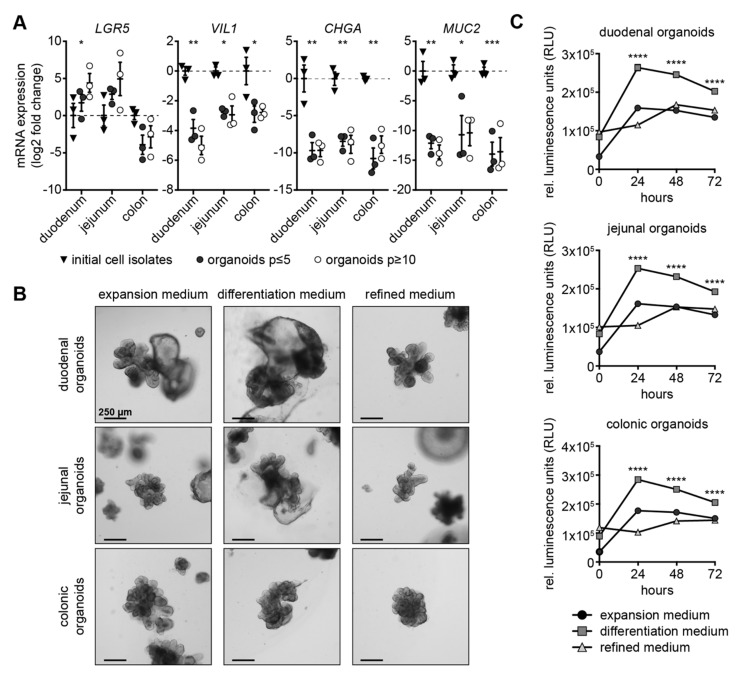
Establishment of new culture conditions for canine intestinal organoids. (**A**) RT-qPCR analysis of initial cell isolates (triangles), organoids with up to five passages (dark circles) and organoids with 10 or more passages (light circles) isolated from duodenum, jejunum or colon using *LGR5*, *VIL1*, *CHGA* and *MUC2* primer. log2 fold changes normalized to expression of initial cell isolates are shown with scatter dot plots; mean is shown; whiskers present SEM; * *p* < 0.05, ** *p* < 0.01 and *** *p* < 0.001, statistical analysis given in detail in Supplementary Tables S2–S4; n = 3 individual dogs. (**B**) Light microscopic images of organoids derived from duodenum, jejunum and colon cultivated in expansion, differentiation and refined medium four days after seeding; scale bars represent 250 µm. (**C**) Mean activity of Annexin V-induced luciferase is shown for 0, 24, 48 and 72 h after addition of substrate to duodenal, jejunal and colonic organoids in expansion (circle), differentiation (square) and refined medium (triangle); whiskers represent SEM; **** *p* < 0.0001; 50 organoids seeded per replicate; n = 6 replicates.

The stem cell marker *LGR5* displayed similar expression levels in low- and high-passage organoids, except high-passage duodenal organoids, which exhibited significant up-regulation of *LGR5* in comparison to their low-passage and initial cell isolate counterparts (Figure 1A). The enterocyte marker villin 1 (*VIL1*) exhibited significantly reduced expression in duodenal, jejunal and colonic organoids in low- and high-passage organoids compared to the initial cell isolates. Enteroendocrine cell marker chromogranin A (*CHGA*) [27] and goblet cell marker mucin 2 (*MUC2*) [28,29] transcripts were barely detectable in organoids when kept in expansion medium throughout passages (Figure 1A).

### 3.2. Canine Intestinal Organoids Grow Efficiently in Refined Medium

To best reflect the in vivo situation and thus allow for downstream applications including drug and toxicity assessment, microbiome studies and modelling of infectious disease, intestinal organoids should harbor stem cells, undifferentiated transit-amplifying cells and differentiated cells. Three different media were compared to identify the ideal medium composition supporting such a scenario: (i) an expansion medium for the culture of canine organoids; (ii) a differentiation medium characterized by omission of nicotinamide and p38-MAPK inhibitor [14]; and (iii) a novel refined medium based on canine expansion medium without nicotinamide, p38-MAPK inhibitor, N2 and EGF, but supplemented with IGF1 and FGF2 as suggested for human organoid culture [25]. After several passages, duodenal, jejunal and colonic canine organoids grown in expansion medium were partly transferred to refined medium for long-term cultivation. For experiments, expansion medium-grown organoids where partly transferred to differentiation medium and after four days, organoids in all three media were harvested (Figure S1A). Growth characteristics and gene expression were assessed. 

Bright field images of duodenal, jejunal and colonic organoids grown in expansion and refined medium displayed intact, budding structures (Figure 1B). In contrast, all organoid types grown in differentiation medium displayed dark and necrotic areas (Figure 1B, Figure S1B). In accordance with these observations, luciferase-based Annexin V assay revealed low and comparable apoptosis in organoids grown in expansion and refined medium, but pronounced apoptosis in organoids grown in differentiation medium (Figure 1C). Organoids in expansion and refined medium could be cultivated for prolonged period of 25 passages after adaptation of growth medium (Figure S1C). Taken together, only expansion and refined medium supported organoid growth.

### 3.3. Refined Medium Promotes Expression of Cell Differentiation Markers in Canine Intestinal Organoids

We next aimed at defining the cellular composition of organoids with respect to the different culture conditions. To this end, total RNA isolated from initial cell isolates and from the three different organoid types cultured in the three different media were subjected to gene expression analysis of stem cell and differentiation markers using RT-qPCR. 

**Figure 2:**
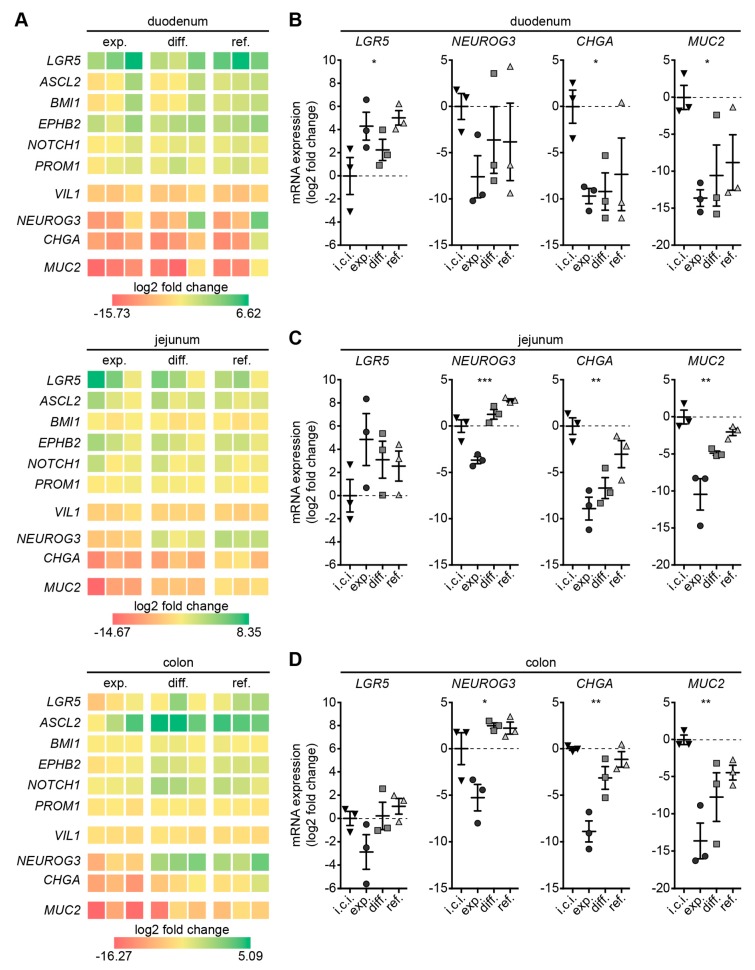
Refined medium and differentiation medium show elevated marker expression of enteroendocrine and goblet cells. (**A**) Heat map of log2 fold changes derived from RT-qPCR data of duodenal, jejunal and colonic organoids cultivated four days in expansion, differentiation and refined medium normalized to initial cell isolates (i.c.i. = 0); data from three individuals are shown in side-by-side columns. (**B**–**D**) Individual scatter dot plots of gene expression data from (**A**) shown for stem cell marker *LGR5*, secretory lineage precursor marker *NEUROG3*, enteroendocrine cell marker *CHGA* and goblet cell marker *MUC2* for organoids derived from duodenum (**B**), jejunum (**C**) and colon (**D**) four days after seeding; mean is shown, whiskers are SEM; * *p* < 0.05, ** *p* < 0.01, *** *p* < 0.001, statistical analysis given in detail in Supplementary Tables S5–S7; n = 3 dogs; i.c.i., initial cell isolates; exp., expansion medium; diff., differentiation medium; ref., refined medium.

In general, a heat map representing log2 fold changes of organoids to respective initial cell isolates showed that transcription of stem cell markers was up-regulated in intestinal organoids grown in all three media. Conversely, transcription of differentiation markers was exclusively up-regulated in intestinal organoids grown in differentiation or refined medium (Figure 2A). 

In more detail, duodenal organoids displayed significant up-regulation of stem cell marker *LGR5* [30,31] gene expression irrespective of culture conditions (Figure 2B). Culture of duodenal organoids in differentiation and refined medium enhanced mRNA expression of the differentiation markers *NEUROG3* (enteroendocrine progenitor cells) [32,33], *CHGA* (enteroendocrine cells) and *MUC2* (Goblet cells). Transcription of stem cell markers *ASCL2*, *EPHB2*, *BMI1*, *NOTCH1* and *PROM1* [30,31] did not vary significantly throughout duodenal organoids analyzed, while enterocyte marker *VIL1* mRNA was significantly down-regulated in duodenal organoids irrespective of culture conditions (Figure S2A). 

Jejunal organoids grown in the three different media exhibited no significant difference in *LGR5*, *ASCL2*, *EPHB2*, *BMI1*, *NOTCH1* or *PROM1* transcription compared to corresponding initial cell isolates (Figure 2C, Figure S2B). *NEUROG3*, *CHGA* and *MUC2* gene expression was significantly down-regulated in jejunal organoids grown in expansion medium. Transcription of differentiation markers *NEUROG3*, *CHGA*, and *MUC2* mRNA was up-regulated when culturing jejunal organoids in refined medium. Expression of *VIL1* was slightly, but significantly, reduced throughout jejunal organoids (Figure S2B). 

In colonic organoids, medium compositions had no significant impact on *LGR5* expression, while *NEUROG3*, *CHGA* and *MUC2* transcription was significantly down-regulated in organoids kept in expansion medium (Figure 2D). Culturing colonic organoids in differentiation and refined medium induced an increase of *CHGA* and *MUC2* expression almost to levels displayed by initial cell isolates. In contrast to duodenal and jejunal organoids, colonic organoids showed aberrant *ASCL2*, *EPHB2*, *BMI1* and *PROM1* transcription in comparison to initial cell isolates, whereas *NOTCH1* expression remained grossly unchanged (Figure S2C). *VIL1* transcription was generally slightly lower expressed in colonic organoids irrespective of culture conditions. 

Marker transcription levels in organoids varied to some extent with respect to the individual dog they were derived from. These variations were less pronounced when cultivating organoids—notably jejunal and colonic organoids—in refined medium (Figure 2, Figure S2). Despite these individual variations the presented data indicate that differentiation and refined medium supported cell differentiation to secretory lineage cells within organoids.

### 3.4. Refined and Differentiation Medium Both Support Organoid Differentiation

**Figure 3:**
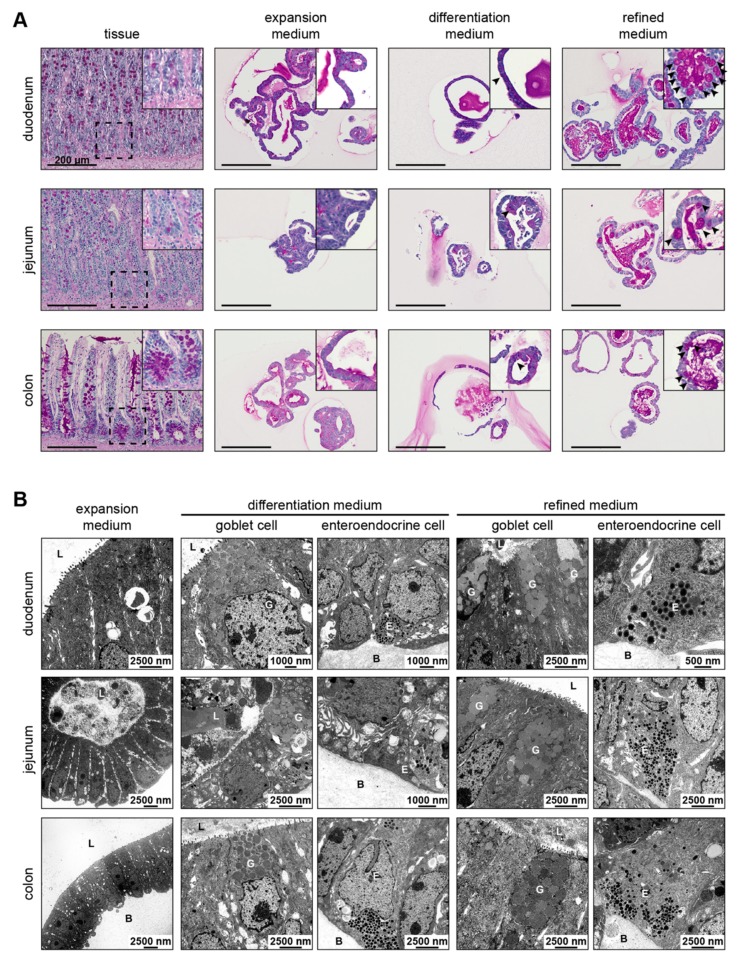
Goblet and enteroendocrine cells are present in organoids upon cultivation in differentiation and refined medium, but not in expansion medium. (**A**) PAS reaction in FFPE sections of canine intestinal tissue or organoids in expansion, differentiation and refined medium derived from duodenum, jejunum and colon; counterstained with Hematoxylin, images of duodenal and jejunal tissue were cropped in order to visualize crypts; scale bars 200 µm, inset depicts higher magnification; arrow heads indicate PAS positive goblet cells; one representative image is shown. (**B**) TEM images of duodenal, jejunal and colonic organoids in expansion, differentiation and refined medium; scale bars are indicated; L, lumen; B, basal lamina; G, goblet cell; E, enteroendocrine cell.

The detection of markers for goblet and enteroendocrine cells by RT-qPCR prompted us to confirm and localize these cells within organoids. Therefore, periodic-acid Schiff (PAS) reaction and transmission electron microscopy (TEM) were used. PAS reaction of mucin as revealed by a purple signal demonstrated the presence of goblet cells that were evenly distributed in duodenal and jejunal tissue sections along the crypt-villus axis, and at higher frequency around the crypt base in colon tissue sections (Figure 3A). In organoid culture, expansion medium failed to induce differentiation into goblet cells, while growth in differentiation and refined medium gave rise to goblet cells irrespective of the intestinal segment. These observations were in accordance with *MUC2* transcription data (see Figure 2) and further substantiated by TEM, which revealed the presence of goblet cells with apical large mucin granules and enteroendocrine cells characterized by small dark basal granules in organoids grown in differentiation and refined medium (Figure 3B and Figure S3B). In contrast, TEM of organoids cultivated in expansion medium revealed uniformly appearing cells harboring microvilli (Figure S3A). These cells were also present in all other conditions suggesting the presence of enterocytes, which is correlating with *VIL1* expression data (see Figure 2 and Figure S2). Interestingly, cell-to-cell interactions such as tight junctions, adherens junctions and desmosomes could be visualized by organoid TEM throughout culture conditions (Figure S3C). These data demonstrate the presence of secretory lineage cells in canine organoids upon cultivation in refined and differentiation medium.

### 3.5. Refined Medium Supports Continuous Growth and Derivation of Organoids

So far, we have been able to verify that organoids in refined medium were differentiating similarly or even better than organoids in differentiation medium (see Figure 2 and Figure 3) without concomitant induction of apoptosis (see Figure 1B,C). Therefore, we conclude that a refined medium composition is beneficial over the use of a separate differentiation medium. In a next step we assessed the effectiveness of refined versus expansion medium on the continuous growth of organoids.

**Figure 4:**
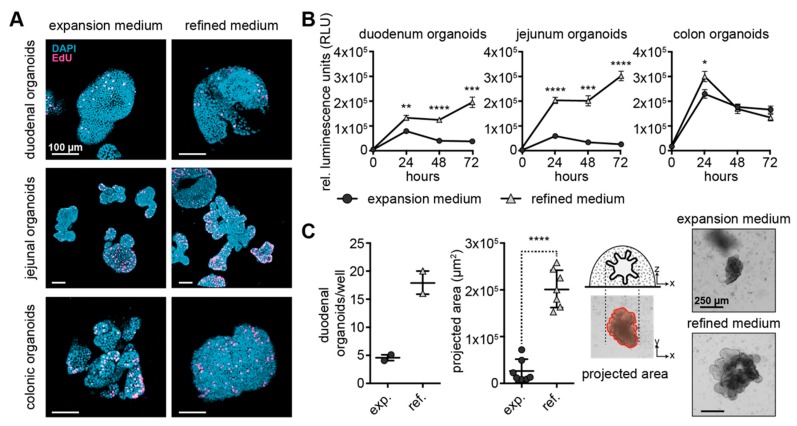
Derivation of organoids is more efficient using refined medium conditions. (**A**) 3D reconstruction of confocal z-stack images from duodenal, jejunal and colonic organoids in expansion and refined medium; staining for EdU after one hour labeling (magenta); organoids counterstained using DAPI (blue); scale bar represents 100 µm. (**B**) Relative luciferase activity of cell viability assay is shown for serial measurements at 0, 24, 48 and 72 h after addition of substrate to duodenal, jejunal and colonic organoids in expansion (circle) and refined medium (triangle); mean is shown; whiskers represent SEM; * *p* < 0.05, ** *p* < 0.01, *** *p* < 0.001 and **** *p* < 0.0001; 50 organoids seeded per replicate; n = 6 replicates. (**C**) Organoid number is shown for freshly isolated duodenal biopsies seeded in expansion and refined medium, in scatter dot plots with mean and range indicated by whiskers, n = 2 wells; projected area of organoids is depicted with mean and SEM defined by whiskers, **** *p* < 0.0001, n = 7 organoids; bright field images of representative organoids are shown, scale bar represents 250 µm.

Importantly, all organoid types showed a similar proliferation pattern, as revealed by a scattered distribution of single, EdU-positive cells within organoids when exposed to the two different medium conditions (Figure 4A). However, viability of duodenal and jejunal organoids in expansion medium was reduced compared to colonic organoids, which could be significantly enhanced by using refined medium (Figure 4B). 

To assess the suitability of refined medium for the establishment of new organoid lines, duodenal biopsies were taken during routine gastroduodenoscopy. Following isolation, starting cell material was cultured either with expansion medium containing Rock inhibitor, or with EGF- and Rock inhibitor-supplemented refined medium for the first two days, then changed to unsupplemented expansion and refined medium. After 10 days in culture, an almost 4-fold higher number of organoids had grown in refined medium compared to in expansion medium (Figure 4C). Organoids exposed to refined medium also displayed a higher projected area compared to organoids formed in expansion medium, indicating that refined medium allows for easier adaption of canine intestinal cells to in vitro culture conditions.

## 4. Discussion

Since the first reports on the derivation of organoids from murine and subsequently from human intestine, the establishment of canine intestinal organoids was a matter of time. Particularly as dogs develop GI diseases like cancer, infectious disease and IBD naturally [23], thereby representing a faithful model system for the corresponding human diseases. In early 2017 our group [17], Kingsbury et al. [34], and Powel and Behnke [16] reported on the successful derivation of canine intestinal organoids. We then focused on advancing organoid cultivation to bring them a step closer to the in vivo situation.

In the presented study, we found that expansion medium does not support stem cell commitment in the secretory lineage using transcription profiling. This finding is supported by reports on human intestinal organoids, which usually harbor only a limited number of differentiated cell types, because they are grown in expansion media that allow for the maintenance of stem cells but do not support secretory lineage differentiation [14,17]. This absence of differentiated cells in expansion medium could not be overcome by prolonged cultivation: low- (≤5) and high-passage (≥10) canine intestinal organoids all tested negative for differentiation marker expression. Recently, Chandra et al. published a well-written paper on the derivation of canine intestinal organoids and the presence of goblet and enteroendocrine cells [35]. The formulation of the expansion medium differs in a 5-fold higher concentration of p38-MAPK inhibitor and the addition of 8% fetal bovine serum FBS (Chandra) compared to the medium formulation used herein. p38-MAPK inhibitor in human organoid culture is used to suppress secretory lineage commitment of intestinal stem cells to prevent their depletion and maintain long-term culture [14,36,37]. The addition of 8% FBS is a likely explanation for the presence of secretory cells, potentially via subsequent activation of p38-MAPK signaling. To create a culture system with defined components, the culture medium used in the study presented herein corresponds to a widely used human organoid medium without FBS [14] that was only supplemented with HGF to allow for the cultivation of over 50 passages. These variations in medium additives may explain the differences between both studies.

To further develop canine intestinal organoid culture, we focused on optimizing the growth medium to obtain a more in vivo-like cell composition with stem cells, enterocytes, goblet and enteroendocrine cells. Recently, a newly composed medium was shown to enable stem cells of human intestinal organoids to undergo differentiation, while concomitantly preserving the stem cell pool [25]. To overcome the intrinsic limitations of classical expansion medium, we adapted our canine intestinal expansion medium based on the suggestions of Fujii et al. [25] by (i) withdrawal of nicotinamide, p38-MAPK inhibitor, N2 and EGF; and (ii) addition of IGF1 and FGF2. This medium termed “refined medium” promoted stem cell growth and differentiation of canine intestinal organoids simultaneously for over 6 months of cultivation (data not shown). To assess the benefits and limitations of this refined medium in regard to organoid growth and lineage commitment, canine intestinal organoids grown in expansion, differentiation or refined medium were comparatively characterized.

While duodenal, jejunal and colonic organoids displayed a viable morphology upon culture in expansion and refined medium, differentiation medium induced apoptosis in all three intestinal segments. Interestingly, apoptosis peaked as early as 24 h after transfer to differentiation medium, indicating that programmed cell death was initiated by differentiation-unrelated factors. This assumption is supported by previous reports on the promoting effect of nicotinamide on cell survival in human pluripotent stem cells [38], on proliferation of aged murine organoids [39] and on inducing apoptosis via reduced intracellular NAD concentrations upon its withdrawal [40]. Taken together, differentiation medium induced apoptosis, thus not supporting long-term culture of canine intestinal organoids. This induction of cell death by the medium formulation is unfavorable when differentiation, proliferation or even apoptosis are investigated upon compound treatment for drug development. In addition, differentiation of organoids in expansion medium must be repeated for every individual experiment making them susceptible to fluctuations between media batches, illustrating the need for a better culture system.

Refined medium induced similar levels of differentiation or even outperformed differentiation medium in this regard. Duodenal, jejunal and colonic organoids grown in refined medium revealed enhanced expression of markers indicating the presence of secretory lineage precursors, enteroendocrine and goblet cells. Interestingly, organoids in refined medium expressed comparable or even higher levels of stem cell marker compared to organoids in expansion medium. Organoids irrespective of the intestinal segment or media composition expressed the enterocyte marker *VIL1*. These data confirm findings reported for human intestinal organoids grown in niche-inspired culture media that supported expression of enteroendocrine, goblet and stem cell markers [25] and data reported for villin1 positive enterocytes in murine intestinal organoids [12]. However, expression of the differentiation markers *CHGA*, *MUC2* and *VIL1* in differentiation and refined medium reached the levels of the i.c.i. only to a certain extent. While an in vitro culture model cannot fully reproduce the complex in vivo situation, our data show that growth conditions in refined medium are more similar compared to those in expansion medium.

Since only a limited number of specific antibodies were available for reliable characterization of canine cells, we used two well-established alternative methods, PAS reaction and TEM, for the detection of goblet cells and enteroendocrine cells. These results supported our RT-qPCR data, substantiating that only organoids grown in differentiation and refined medium harbored enteroendocrine and goblet cells. These findings are in accordance with data obtained for human intestinal organoids grown in differentiation [14] or niche-inspired medium [25]. Furthermore, the presence of enterocytes in organoids cultivated in expansion, differentiation and refined medium of all segments was supported by microvilli bearing cells as reported for murine intestinal organoids [12], indicating that enterocytes are present irrespective of culture condition. 

So far, our data provided evidence that cultivation of canine intestinal organoids in refined medium is preferable to the use of differentiation medium, since it promotes stem cell growth and differentiation simultaneously, allows long-term growth without fluctuations between media batches and does not induce apoptosis. Therefore, further analysis focused on comparison of organoid growth in expansion and refined medium.

The comparison of proliferative behavior differences between canine intestinal organoids grown in expansion versus refined medium by EdU incorporation assays revealed similar proliferation levels for all organoid types irrespective of the medium used. Yet, viability data of duodenal and jejunal organoids indicate that expansion medium does not support small intestinal organoid cultivation as effective as refined medium. The colonic epithelium is highly populated with various bacteria and has less digestive and absorptive function than small intestinal epithelium possibly resulting in more robustness with respect to cultivation. Therefore, colonic organoids can be expanded in both expansion and refined medium efficiently.

Importantly, generation of organoids from duodenal biopsies was more successful in refined than expansion medium. Not only the number of organoids per well was enhanced, but also their projected area. This feature has an important impact on canine intestinal disease modelling, especially when donor tissue stems from GI enteropathies with severely affected epithelium. The high suitability of refined medium for canine organoid establishment also matches the higher plating efficacy shown for human single-cell dissociated organoids grown in niche-inspired media [25]. 

## 5. Conclusions

Taken together, we strongly recommend the use of refined medium for establishment and long-term culture of canine intestinal organoids. We could clearly show that this medium sustains stem cell growth, while simultaneously promoting differentiation of stem cells/immature cells into enterocytes, enteroendocrine and goblet cells. Canine intestinal organoids cultivated in refined medium bear the advantage of an easy to handle, reproducible and stable culture system, thereby representing a physiologically superior in vitro system for disease modelling, drug development, toxicity studies and personalized medicine. Compared to conventional expansion and differentiation media, the refined medium presented herein allows for more accurate assessment of genetic and epigenetic impacts on canine intestinal cell differentiation, bringing organoids a step closer to the in vivo situation.

## Figures and Tables

**Table 1:** List of media compositions used in this study. Basal medium consists of Advanced DMEM/F12, 2 mM GlutaMAX, 10 mM HEPES, 1x B27, 1 mM N-acetylcystein. Final concentrations are given.

Reagent	Expansion Medium	Differentiation Medium	Refined Medium
basal medium	+	+	+
N2	1x	1x	−
EGF	50 ng/mL	50 ng/mL	− ^a^
Noggin	100 ng/mL	100 ng/mL	100 ng/mL
Rspo1	10% *v*/*v*	10% *v*/*v*	10% *v*/*v*
Wnt3a	43% *v*/*v*	43% *v*/*v*	50% *v*/*v*
Nicotinamide	10 mM	−	−
Gastrin	10 nM	10 nM	10 nM
A83-01	500 nM	500 nM	500 nM
SB202190	10 µM	−	−
HGF	50 ng/mL	50 ng/mL	50 ng/mL
IGF1	−	−	100 ng/mL
FGF2	−	−	50 ng/mL

^a^ in refined medium 50 ng/mL EGF is only added for the first two days after isolation or medium adaption.

## Supplementary Materials

The following are available online at https://www.mdpi.com/2073-4409/9/4/822/s1, Figure S1: Cultivation scheme of organoids and images of organoids in differentiation medium and after prolonged cultivation in expansion and refined medium; Figure S2: Gene expression of stem cell marker is increased in colonic organoids; Figure S3: TEM images of microvilli and cell-cell junctions in all conditions and goblet cells in organoids cultivated in differentiation and refined medium; Table S1: qPCR primer specifications used in this study; Table S2: Statistical analysis of duodenal tissue vs. duodenal organoids; Table S3: Statistical analysis of jejunal tissue vs. jejunal organoids; Table S4: Statistical analysis of colonic tissue vs. colonic organoids; Table S5: Statistical analysis of duodenal organoids in different media; Table S6: Statistical analysis of jejunal organoids in different media; Table S7: Statistical analysis of colonic organoids in different media.
