# Supplementary material for: Generation of Differentiating and Long-Living Intestinal Organoids Reflecting the Cellular Diversity of Canine Intestine
Source: Cells. 2020 Mar 28;9(4):822. doi: 10.3390/cells9040822 (PMC7226743; doi:10.3390/cells9040822)
Supplement: Supplementary file 1 [file cells-09-00822-s001.zip › Supplementary Figures_revised.pdf]

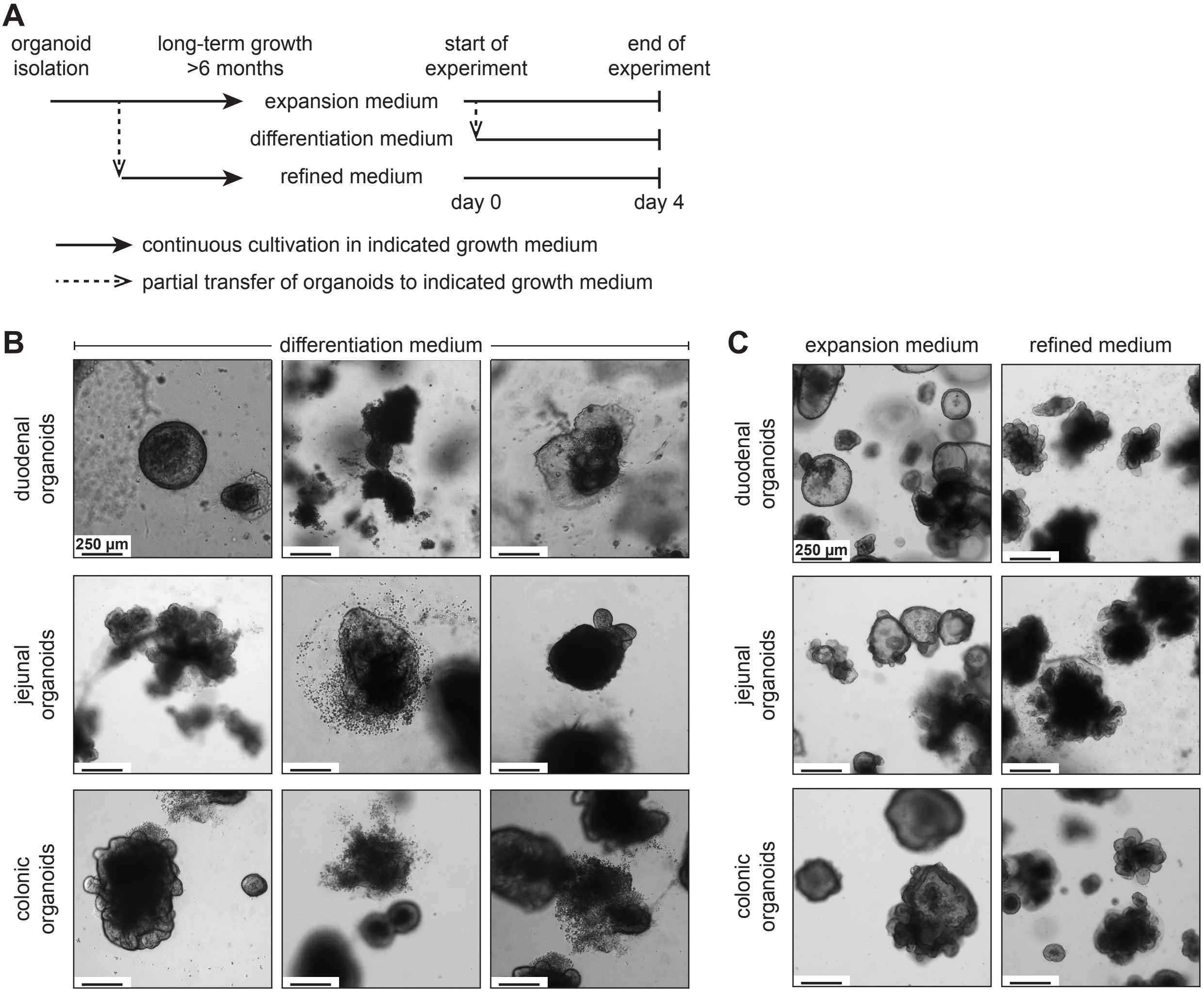

Figure S1. Cultivation scheme of organoids and images of organoids in differentiation medium and after prolonged cultivation in expansion and refined medium.
(A) Outline of cultivation scheme. Duodenal, jejunal and colonic organoids were initially isolated using expansion medium and were partially transferred to refined medium for long-term cultivation; for experiments organoids grown in expansion medium were partially transferred to differentiation medium and cultivated in parallel with expansion and refined medium until harvesting for various assays four days afterwards. **(B)** Light microscopic images of duodenal, jejunal and colonic organoids in differentiation medium from various experiments four days after seeding; scale bar represents 250 µm. **(C)** Light microscopic images of organoids derived from duodenum, jejunum and colon cultivated for 25 passages in refined medium and expansion medium four days after seeding; scale bars represent 250 µm.

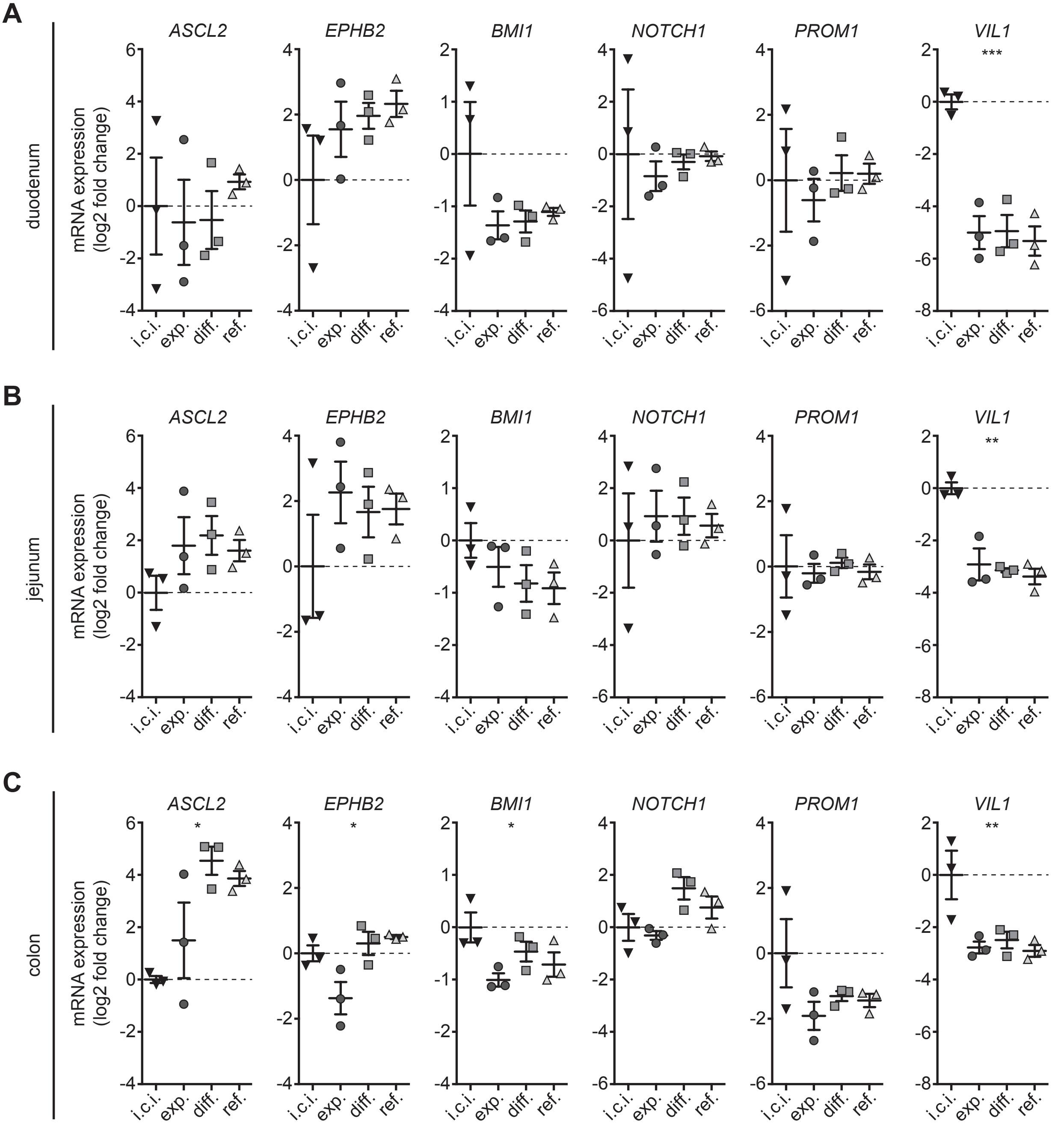

Figure S2. Gene expression of stem cell marker is increased in colonic organoids. (A-C) Individual scatter dot plots of gene expression data from Figure 2A shown for stem cell marker *ASCL2*, *EPHB2*, *NOTCH1*, *BMI1*, *PROM1* and the enterocyte marker *VIL1* for organoids derived from duodenum **(A)**, jejunum **(B)** and colon **(C)** four days after seeding; mean is shown, whiskers are SEM; * $p<0.05$, ** $p<0.01$ and *** $p<0.001$, statistical analysis given in detail in Supplementary Table S5-S7; n=3 dogs; i.c.i., initial cell isolates; exp., expansion medium; diff., differentiation medium; ref., refined medium.

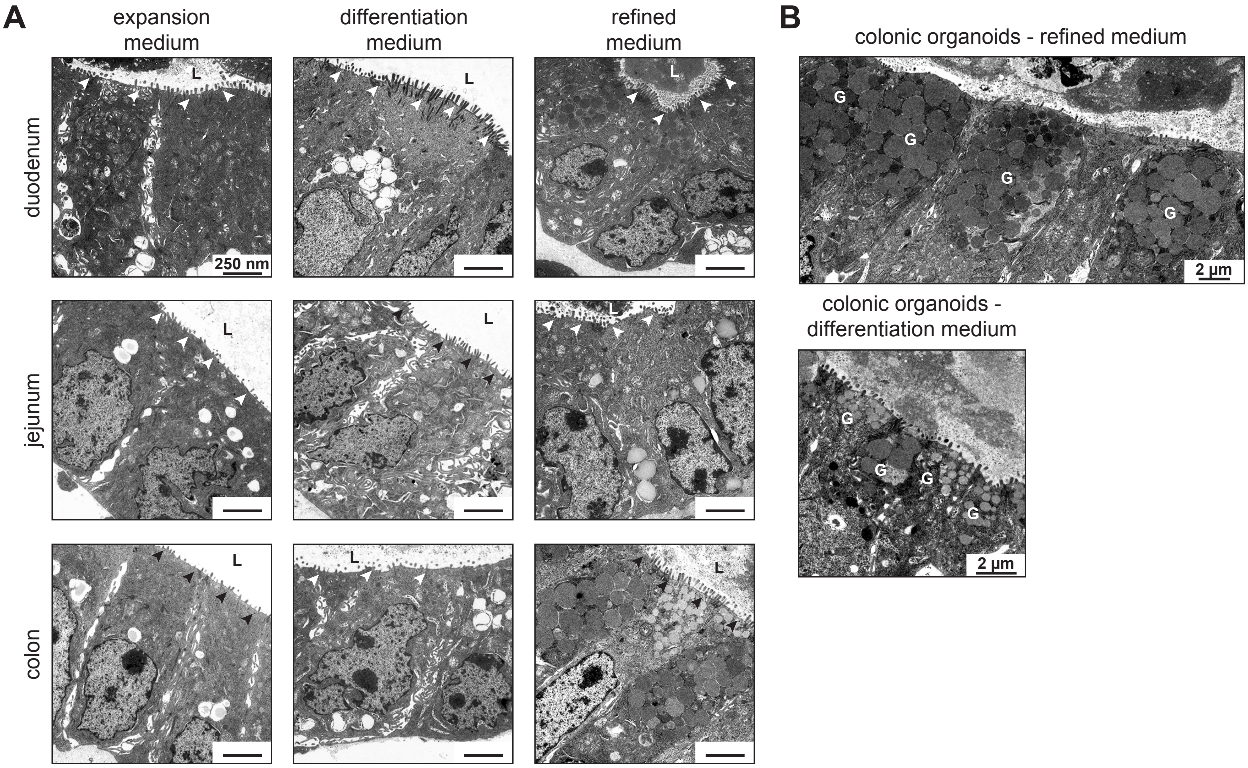

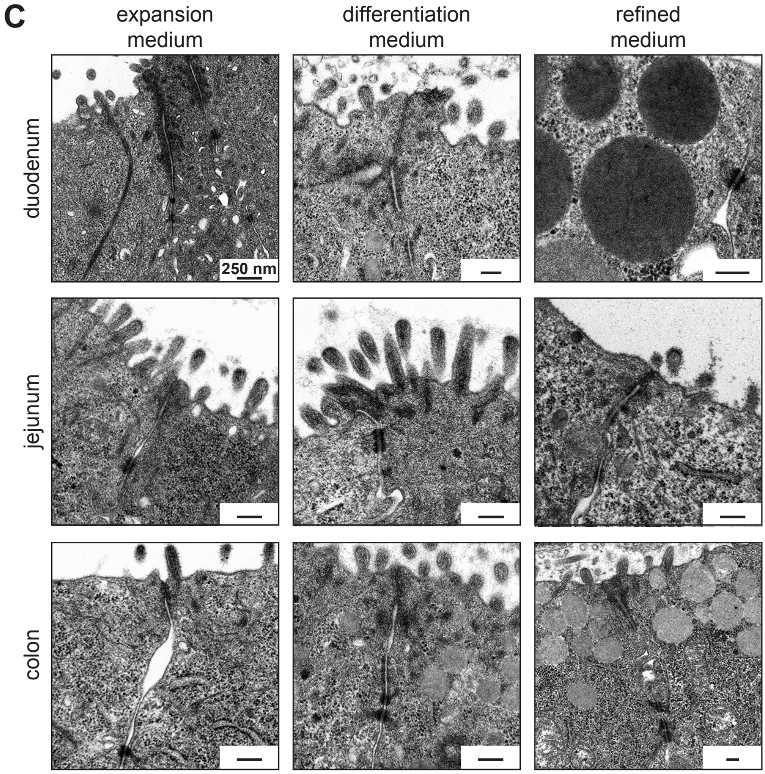

Figure S3. TEM images of microvilli and cell-cell junctions in all conditions and goblet cells in organoids cultivated in differentiation and refined medium.
(A) Respresentative TEM images of microvilli (arrowheads) in organoids of all conditions from Figure 3B; L, lumen; scale bar represents 250 nm. **(B)** Representative TEM images of colonic organoids in differentiation and refined medium from Figure 3B; G, goblet cells; scale bar represents 2 µm. **(C)** TEM images depicting tight junctions, adherens junctions and desmosomes of organoids from Figure 3B; scale bars represent 250 nm.
