# Supplementary material for: Generation of Differentiating and Long-Living Intestinal Organoids Reflecting the Cellular Diversity of Canine Intestine
Source: Cells. 2020 Mar 28;9(4):822. doi: 10.3390/cells9040822 (PMC7226743; doi:10.3390/cells9040822)
Supplement: Supplementary file 1 [file cells-09-00822-s001.zip › Supplementary tables1to7.pdf]

Table S1: qPCR primer specifications used in this study

A summary of all primer sequences that were used, including GeneBank accession numbers and amplicon sizes.

Gene	GeneBank Accession №	5'-3' primer sequence	Amplicon size (bp)
ASCL2	XM_005631346.3	F: GTGAAGCTGGTGAACTTGG<br>R: CACCTTACTCAGCTTCTTGC	81
BMI1	NM_001287063.1	F: GAACAACCAGAATCAAGATCAC<br>R: GGTCTCCAAGTAACGCAC	140
CHGA	XR_002628933.1	F: GGTCCTGGCTCTGTTGTTCC<br>R: CTCGCGAGAAAAGACAACCG	196
EPHB2	XM_005617823	F: CTGCCACTCATCATCGGCTC<br>R: CCCCCGTCTTCTGTTACACAC	90
HPRT	NM_001003357.2	F: CCCCAGCGTCGTGATTAGTG<br>R: ACACTTTTTCCAAATCCTCAGCG	92
LGR5	XM_014117119.2	F: CTCAGCGTCTTCACCTCCT<br>R: TGGGAATGTATGTCAAAGCGT	130
MUC2	XM_022405513.1	F: TCCTCTACCCTCGTCTACTGC<br>R: GACGGGCATGACCAGTTGAA	149
NEUROG3	XM_546140.2	F: AAAGGCCGGCTCATCCTAAC<br>R: TCGTAGGTCCCTTGGACAGT	116
NOTCH1	XM_005625433.1	F: TACCGGCCAGAACTGTGAGGAGAA<br>R: GGAGGGCAGCGGCAGTTGTAAGTA	108
PROM1	NM_001314109.1	F: CTGGGGCTGCTCTTTGTGAT<br>R: AGGCCCCATTTTTCTTCTGTC	115
VIL1	XM_545642.5	F: TGCCAACACCAAGAGATTGC<br>R: GCAGGAACACATCGTCCTCT	143

Table S2: Statistical analysis of duodenal tissue vs. duodenal organoids
Two-way ANOVA, Tukey multiple comparisons of mean, 95 % family-wise confidence level; i.c.i., initial cell isolates; p≥10, organoids in passage higher or equal 10; p≤5, organoids in passage less or equal 5; code: 0 *** 0.001 ** 0.01 * 0.05 . 0.1

LGR5	**Df**	**Sum Sq**	**Mean Sq**	**F value**	**Pr(>F)**	
**Medium**	2	28.7511	14.3755	13.271	0.01715	*
**Individuals**	2	27.6783	13.8392	12.776	0.01832	*
**Residuals**	4	4.3329	1.0832			

multiple comparisons - Medium

LGR5	**diff**	**lwr**	**upr**	**p adj**
**p≥10-i.c.i.**	4.342488	1.313815	7.3711615	0.0151270
**p≤5-i.c.i.**	1.688923	-1.339750	4.7175961	0.2305607
**p≤5-p≥10**	-2.653565	-5.682239	0.3751076	0.0743799

multiple comparisons - Individuals

LGR5	**diff**	**lwr**	**upr**	**p adj**
**#2-#1**	2.986453	-0.04221994	6.015126	0.0522168
**#3-#1**	4.167174	1.13850073	7.195847	0.0174639
**#3-#2**	1.180721	-1.84795239	4.209394	0.4271312

VIL1	**Df**	**Sum Sq**	**Mean Sq**	**F value**	**Pr(>F)**	
**Medium**	2	40.755	20.3777	44.219	0.001873	**
**Individuals**	2	3.219	1.6097	3.493	0.132570	
**Residuals**	4	1.843	0.4608			

multiple comparisons - Medium

VIL1	**diff**	**lwr**	**upr**	**p adj**
**p≥10-i.c.i.**	-4.967003	-6.9424487	-2.991557	0.0019050
**p≤5-i.c.i.**	-3.852572	-5.8280183	-1.877126	0.0049694
**p≤5-p≥10**	1.114430	-0.8610156	3.089876	0.2250584

multiple comparisons - Individuals

VIL1	**diff**	**lwr**	**upr**	**p adj**
**#2-#1**	-0.028771	-2.0042166	1.946675	0.9985162
**#3-#1**	1.254108	-0.7213381	3.229554	0.1732968
**#3-#2**	1.282879	-0.6925675	3.258325	0.1642985

Table S2 (continued)

CHGA	Df	Sum Sq	Mean Sq	F value	Pr(>F)	
**Medium**	2	186.757	93.379	18.7766	0.009266	**
**Individuals**	2	9.771	4.885	0.9823	0.449727	
**Residuals**	4	19.893	4.973			

multiple comparisons - Medium

CHGA	diff	lwr	upr	p adj
**p≥10-i.c.i.**	-9.67218414	-16.161613	-3.182755	0.0131975
**p≤5-i.c.i.**	-9.65431161	-16.143740	-3.164883	0.0132841
**p≤5-p≥10**	0.01787253	-6.471556	6.507301	0.9999469

multiple comparisons - Individuals

CHGA	diff	lwr	upr	p adj
**#2-#1**	1.162652	-5.326777	7.652081	0.8083221
**#3-#1**	2.548925	-3.940504	9.038353	0.4227062
**#3-#2**	1.386273	-5.103156	7.875701	0.7437583

MUC2	Df	Sum Sq	Mean Sq	F value	Pr(>F)	
**Medium**	2	332.64	166.322	26.238	0.00502	**
**Individuals**	2	3.44	1.718	0.271	0.77558	
**Residuals**	4	25.36	6.339			

multiple comparisons - Medium

MUC2	diff	lwr	upr	p adj
**p≥10-i.c.i.**	-13.573963	-20.90050	-6.247423	0.0060086
**p≤5-i.c.i.**	-12.090983	-19.41752	-4.764444	0.0091676
**p≤5-p≥10**	1.482979	-5.84356	8.809519	0.7650940

multiple comparisons - Individuals

MUC2	diff	lwr	upr	p adj
**#2-#1**	1.2526722	-6.073867	8.579212	0.8230733
**#3-#1**	1.3618028	-5.964737	8.688342	0.7960235
**#3-#2**	0.1091306	-7.217409	7.435670	0.9984481

Table S3: Statistical analysis of jejunal tissue vs. jejunal organoids
Two-way ANOVA, Tukey multiple comparisons of mean, 95 % family-wise confidence level; i.c.i., initial cell isolates; p≥10, organoids in passage higher or equal 10; p≤5, organoids in passage less or equal 5; code: 0 *** 0.001 ** 0.01 * 0.05 . 0.1

LGR5	**Df**	**Sum Sq**	**Mean Sq**	**F value**	**Pr(>F)**
**Medium**	2	35.433	17.7163	2.1792	0.2290
**Individuals**	2	11.750	5.8751	0.7227	0.5396
**Residuals**	4	32.518	8.1296		

multiple comparisons - Medium

LGR5	**diff**	**lwr**	**upr**	**p adj**
**p≥10-i.c.i.**	4.841921	-3.455141	13.138983	0.2093951
**p≤5-i.c.i.**	2.785817	-5.511245	11.082879	0.5149856
**p≤5-p≥10**	-2.056104	-10.353166	6.240958	0.6778530

multiple comparisons - Individuals

LGR5	**diff**	**lwr**	**upr**	**p adj**
**#2-#1**	1.311090	-6.985972	9.608153	0.8457849
**#3-#1**	-1.485920	-9.782983	6.811142	0.8084512
**#3-#2**	-2.797011	-11.094073	5.500051	0.5126569

VIL1	**Df**	**Sum Sq**	**Mean Sq**	**F value**	**Pr(>F)**	
**Medium**	2	15.8513	7.9257	14.5999	0.01452	*
**Individuals**	2	0.5618	0.2809	0.5174	0.63116	
**Residuals**	4	32.518	8.1296			

multiple comparisons - Medium

VIL1	**diff**	**lwr**	**upr**	**p adj**
**p≥10-i.c.i.**	-2.901538	-5.045581	-0.757494	0.0184941
**p≤5-i.c.i.**	-2.720210	-4.864254	-0.576167	0.0230619
**p≤5-p≥10**	0.181327	-1.962716	2.325371	0.9517448

multiple comparisons - Individuals

VIL1	**diff**	**lwr**	**upr**	**p adj**
**#2-#1**	0.005176	-2.138868	2.149220	0.9999592
**#3-#1**	0.532565	-1.611479	2.676608	0.6767305
**#3-#2**	0.527389	-1.616655	2.671432	0.6813906

Table S3 (continued)

CHGA	Df	Sum Sq	Mean Sq	F value	Pr(>F)	
**Medium**	2	150.582	75.291	40.6131	0.00220	**
**Individuals**	2	9.011	4.505	2.4303	0.20379	
**Residuals**	4	7.415	1.854			

multiple comparisons - Medium

CHGA	diff	lwr	upr	p adj
**p≥10-i.c.i.**	-8.8779275	-12.840066	-4.915789	0.0029528
**p≤5-i.c.i.**	-8.4611420	-12.423281	-4.499003	0.0035397
**p≤5-p≥10**	0.4167855	-3.545353	4.378924	0.9268206

multiple comparisons - Individuals

CHGA	diff	lwr	upr	p adj
**#2-#1**	2.4213708	-1.540768	6.383510	0.1891011
**#3-#1**	0.8817167	-3.080422	4.843856	0.7266449
**#3-#2**	-1.5396541	-5.501793	2.422485	0.4290366

MUC2	Df	Sum Sq	Mean Sq	F value	Pr(>F)	
**Medium**	2	222.841	111.421	12.9272	0.01795	*
**Individuals**	2	60.170	30.085	3.4905	0.13269	
**Residuals**	4	34.476	8.619			

multiple comparisons - Medium

MUC2	diff	lwr	upr	p adj
**p≥10-i.c.i.**	-10.4130909	-18.956319	-1.869863	0.0263949
**p≤5-i.c.i.**	-10.6925517	-19.235780	-2.149324	0.0241477
**p≤5-p≥10**	-0.2794608	-8.822689	8.263767	0.9925488

multiple comparisons - Individuals

MUC2	diff	lwr	upr	p adj
**#2-#1**	6.198464	-2.344764	14.741692	0.1248425
**#3-#1**	1.972508	-6.570720	10.515736	0.7105466
**#3-#2**	-4.225956	-12.769184	4.317272	0.2914304

Table S4: Statistical analysis of colonic tissue vs. colonic organoids
Two-way ANOVA, Tukey multiple comparisons of mean, 95 % family-wise confidence level; i.c.i., initial cell isolates; p≥10, organoids in passage higher or equal 10; p≤5, organoids in passage less or equal 5; code: 0 *** 0.001 ** 0.01 * 0.05 . 0.1

LGR5	**Df**	**Sum Sq**	**Mean Sq**	**F value**	**Pr(>F)**
**Medium**	2	25.3744	12.6872	3.2763	0.1437
**Individuals**	2	9.6454	4.8227	1.2454	0.3798
**Residuals**	4	15.4897	3.8724		

multiple comparisons - Medium

LGR5	**diff**	**lwr**	**upr**	**p adj**
**p≥10-i.c.i.**	-2.873601	-8.600003	2.852801	0.2837841
**p≤5-i.c.i.**	-3.985140	-9.711542	1.741262	0.1387964
**p≤5-p≥10**	-1.111539	-6.837941	4.614863	0.7807217

multiple comparisons - Individuals

LGR5	**diff**	**lwr**	**upr**	**p adj**
**#2-#1**	1.9765268	-3.749875	7.702929	0.4988967
**#3-#1**	2.3640237	-3.362378	8.090426	0.3934388
**#3-#2**	0.3874969	-5.338905	6.113899	0.9686938

VIL1	**Df**	**Sum Sq**	**Mean Sq**	**F value**	**Pr(>F)**	
**Medium**	2	16.0401	8.0201	7.2596	0.04665	*
**Individuals**	2	2.8117	1.4058	1.2725	0.37350	
**Residuals**	4	4.4190	1.1047			

multiple comparisons - Medium

VIL1	**diff**	**lwr**	**upr**	**p adj**
**p≥10-i.c.i.**	-2.767718	-5.826313	0.290878	0.0676692
**p≤5-i.c.i.**	-2.892125	-5.950721	0.166471	0.0593407
**p≤5-p≥10**	-0.124408	-3.183004	2.934188	0.9885146

multiple comparisons - Individuals

VIL1	**diff**	**lwr**	**upr**	**p adj**
**#2-#1**	0.8519800	-2.206616	3.910576	0.6189954
**#3-#1**	-0.5021408	-3.560737	2.556455	0.8351045
**#3-#2**	-1.3541208	-4.412717	1.704475	0.3529565

Table S4 (continued)

CHGA	Df	Sum Sq	Mean Sq	F value	Pr(>F)	
**Medium**	2	198.334	99.167	44.6617	0.00184	**
**Individuals**	2	11.148	5.574	2.5103	0.19663	
**Residuals**	4	8.882	2.220			

multiple comparisons - Medium

CHGA	diff	lwr	upr	p adj
**p≥10-i.c.i.**	-8.899055	-13.23523	-4.562882	0.0041088
**p≤5-i.c.i.**	-10.756042	-15.09221	-6.419869	0.0020062
**p≤5-p≥10**	-1.856987	-6.19316	2.479186	0.3720792

multiple comparisons - Individuals

CHGA	diff	lwr	upr	p adj
**#2-#1**	1.842330	-2.493843	6.178502	0.3766735
**#3-#1**	-0.819035	-5.155208	3.517138	0.7904525
**#3-#2**	-2.661364	-6.997537	1.674808	0.1872744

MUC2	Df	Sum Sq	Mean Sq	F value	Pr(>F)	
**Medium**	2	379.57	189.786	73.2049	0.00071	***
**Individuals**	2	49.15	24.577	9.4799	0.03035	*
**Residuals**	4	10.37	2.593			

multiple comparisons - Medium

MUC2	diff	lwr	upr	p adj
**p≥10-i.c.i.**	-13.5861904	-18.271661	-8.900720	0.0011010
**p≤5-i.c.i.**	-13.9588393	-18.644309	-9.273369	0.0009916
**p≤5-p≥10**	-0.3726488	-5.058119	4.312821	0.9571425

multiple comparisons - Individuals

MUC2	diff	lwr	upr	p adj
**#2-#1**	5.2510127	0.5655425	9.936483	0.0348169
**#3-#1**	0.6513824	-4.0340877	5.336853	0.8773299
**#3-#2**	-4.5996302	-9.2851004	0.085840	0.0529372

Table S5: Statistical analysis of duodenal organoids in different media
Two-way ANOVA, Tukey multiple comparisons of mean, 95 % family-wise confidence level; i.c.i., initial cell isolates; Exp., expansion medium; Diff., differentiation medium; Ref., refined medium; code: 0 *** 0.001 ** 0.01 * 0.05 . 0.1

LGR5	**Df**	**Sum Sq**	**Mean Sq**	**F value**	**Pr(>F)**	
**Medium**	3	46.631	15.5436	5.5390	0.03653	*
**Individuals**	2	15.264	7.6319	2.7196	0.14430	
**Residuals**	6	16.837	2.8062			

multiple comparisons - Medium

LGR5	**diff**	**lwr**	**upr**	**p adj**
**Diff.-i.c.i.**	2.2729562 -	2.4618836	7.007796	0.4159926
**Exp.-i.c.i.**	4.3424884 -	0.3923513	9.077328	0.0698991
**Ref.-i.c.i.**	5.0589521	0.3241124	9.793792	0.0381549
**Exp.-Diff.**	2.0695323 -	2.6653075	6.804372	0.4854950
**Ref.-Diff.**	2.7859960 -	1.9488438	7.520836	0.2730038
**Ref.-Exp.**	0.7164637 -	4.0183761	5.451303	0.9502100

multiple comparisons - Individuals

LGR5	**diff**	**lwr**	**upr**	**p adj**
**#2-#1**	1.446813	-2.1876418	5.081268	0.4844163
**#3-#1**	2.761534	-0.8729209	6.395989	0.1265886
**#3-#2**	1.314721	-2.3197339	4.949176	0.5429705

NEUROG3	**Df**	**Sum Sq**	**Mean Sq**	**F value**	**Pr(>F)**	
**Medium**	3	86.881	28.960	4.4266	0.05768	.
**Individuals**	2	186.133	93.067	14.2254	0.00528	**
**Residuals**	6	39.254	6.542			

multiple comparisons - Medium

NEUROG3	**diff**	**lwr**	**upr**	**p adj**
**Diff.-i.c.i.**	-3.6017701	-10.831300	3.6277601	0.3887233
**Exp.-i.c.i.**	-7.6066291	-14.836159	-0.377099	0.0406678
**Ref.-i.c.i.**	-3.7967634	-11.026294	3.4327668	0.3506542
**Exp.-Diff.**	-4.0048589	-11.234389	3.2246713	0.3133035
**Ref.-Diff.**	-0.1949933	-7.424524	7.0345369	0.9996744
**Ref.-Exp.**	3.8098656	-3.419665	11.039396	0.3482026

multiple comparisons - Individuals

NEUROG3	**diff**	**lwr**	**upr**	**p adj**
**#2-#1**	2.276278	-3.273096	7.825653	0.4657066
**#3-#1**	9.256877	3.707502	14.806252	0.0052379
**#3-#2**	6.980598	1.431224	12.529973	0.0195958

Table S5 (continued)

CHGA	Df	Sum Sq	Mean Sq	F value	Pr(>F)	
**Medium**	3	180.728	60.243	5.6176	0.03546	*
**Individuals**	2	75.413	37.707	3.5161	0.09759	.
**Residuals**	6	64.344	10.724			

multiple comparisons - Medium

CHGA	diff	lwr	upr	p adj
**Diff.-i.c.i.**	-9.1954013	-18.451385	0.0605824	0.0513257
**Exp.-i.c.i.**	-9.6721841	-18.928168	-0.4162005	0.0418345
**Ref.-i.c.i.**	-7.3206562	-16.576640	1.9353275	0.1178789
**Exp.-Diff.**	-0.4767829	-9.732767	8.7792008	0.9977609
**Ref.-Diff.**	1.8747451	-7.381239	11.130729	0.8930940
**Ref.-Exp.**	2.3515279	-6.904456	11.607512	0.8156819

multiple comparisons - Individuals

CHGA	diff	lwr	upr	p adj
**#2-#1**	1.820086	-5.284791	8.924963	0.7245827
**#3-#1**	5.988966	-1.115911	13.093843	0.0914564
**#3-#2**	4.168880	-2.935997	11.273757	0.2476559

MUC2	Df	Sum Sq	Mean Sq	F value	Pr(>F)	
**Medium**	3	305.97	101.990	5.7577	0.03365	*
**Individuals**	2	103.46	51.732	2.9204	0.13011	
**Residuals**	6	106.28	17.714			

multiple comparisons - Medium

MUC2	diff	lwr	upr	p adj
**Diff.-i.c.i.**	-10.550415	-22.446420	1.34559	0.0791361
**Exp.-i.c.i.**	-13.573963	-25.469968	-1.67795	0.0288448
**Ref.-i.c.i.**	-8.791213	-20.687218	3.10479	0.1464620
**Exp.-Diff.**	-3.023548	-14.919553	8.87245	0.8154995
**Ref.-Diff.**	1.759202	-10.136803	13.65520	0.9532240
**Ref.-Exp.**	4.782750	-7.113255	16.67875	0.5466663

multiple comparisons - Individuals

MUC2	diff	lwr	upr	p adj
**#2-#1**	1.293926	-7.837427	10.42528	0.9026701
**#3-#1**	6.774234	-2.357119	15.90559	0.1358137
**#3-#2**	5.480308	-3.651045	14.61166	0.2353544

Table S5 (continued)

ASCL2	Df	Sum Sq	Mean Sq	F value	Pr(>F)	
**Medium**	3	4.490	1.4966	0.9419	0.47710	
**Individuals**	2	34.823	17.4115	10.9584	0.00993	**
**Residuals**	6	9.533	1.5889			

multiple comparisons - Medium

ASCL2	diff	lwr	upr	p adj
**Diff.-i.c.i.**	-0.51337033	-4.076165	3.049424	0.9564261
**Exp.-i.c.i.**	-0.60777646	-4.170571	2.955018	0.9313562
**Ref.-i.c.i.**	0.93403075	-2.628764	4.496825	0.8020943
**Exp.-Diff.**	-0.09440612	-3.657201	3.468388	0.9996912
**Ref.-Diff.**	1.44740108	-2.115393	5.010196	0.5391423
**Ref.-Exp.**	1.54180720	-2.020987	5.104602	0.4928633

multiple comparisons - Individuals

ASCL2	diff	lwr	upr	p adj
**#2-#1**	1.343194	-1.39160066	4.077989	0.3525672
**#3-#1**	4.092928	1.35813351	6.827723	0.0088649
**#3-#2**	2.749734	0.01493934	5.484529	0.0489795

EPHB2	Df	Sum Sq	Mean Sq	F value	Pr(>F)
**Medium**	3	9.2193	3.0731	2.0870	0.2034
**Individuals**	2	8.6206	4.3103	2.9272	0.1297
**Residuals**	6	8.8350	1.4725		

multiple comparisons - Medium

EPHB2	diff	lwr	upr	p adj
**Diff.-i.c.i.**	1.9359050	-1.493918	5.365728	0.3005477
**Exp.-i.c.i.**	1.5235298	-1.906294	4.953353	0.4735326
**Ref.-i.c.i.**	2.3040691	-1.125754	5.733892	0.1938990
**Exp.-Diff.**	-0.4123753	-3.842199	3.017448	0.9736473
**Ref.-Diff.**	0.3681640	-3.061659	3.797987	0.9808879
**Ref.-Exp.**	0.7805393	-2.649284	4.210363	0.8576115

multiple comparisons - Individuals

EPHB2	diff	lwr	upr	p adj
**#2-#1**	0.8831013	-1.7496252	3.515828	0.5871956
**#3-#1**	2.0687593	-0.5639671	4.701486	0.1143458
**#3-#2**	1.1856580	-1.4470684	3.818384	0.4068548

Table S5 (continued)

BMI1	Df	Sum Sq	Mean Sq	F value	Pr(>F)
**Medium**	3	3.6514	1.21715	2.1033	0.2012
**Individuals**	2	3.1345	1.56723	2.7082	0.1452
**Residuals**	6	3.4721	0.57869		

multiple comparisons - Medium

BMI1	diff	lwr	upr	p adj
**Diff.-i.c.i.**	-1.28911307	-3.439260	0.8610340	0.2609643
**Exp.-i.c.i.**	-1.36785865	-3.518006	0.7822884	0.2246685
**Ref.-i.c.i.**	-1.10915260	-3.259300	1.0409944	0.3634805
**Exp.-Diff.**	-0.07874558	-2.228893	2.0714015	0.9991882
**Ref.-Diff.**	0.17996047	-1.970187	2.3301075	0.9906683
**Ref.-Exp.**	0.25870605	-1.891441	2.4088531	0.9735930

multiple comparisons - Individuals

BMI1	diff	lwr	upr	p adj
**#2-#1**	0.8203268	-0.8301223	2.470776	0.3452119
**#3-#1**	1.2291397	-0.4213094	2.879589	0.1342980
**#3-#2**	0.4088129	-1.2416362	2.059262	0.7391612

NOTCH1	Df	Sum Sq	Mean Sq	F value	Pr(>F)
**Medium**	3	1.2729	0.4243	0.1085	0.9520
**Individuals**	2	16.0782	8.0391	2.0565	0.2088
**Residuals**	6	23.4544	3.9091		

multiple comparisons - Medium

NOTCH1	diff	lwr	upr	p adj
**Diff.-i.c.i.**	-0.26720075	-5.855523	5.321121	0.9982048
**Exp.-i.c.i.**	-0.82850535	-6.416827	4.759816	0.9529003
**Ref.-i.c.i.**	-0.06704766	-5.655370	5.521274	0.9999712
**Exp.-Diff.**	-0.56130460	-6.149626	5.027017	0.9841946
**Ref.-Diff.**	0.20015309	-5.388169	5.788475	0.9992404
**Ref.-Exp.**	0.76145769	-4.826864	6.349780	0.9626372

multiple comparisons - Individuals

NOTCH1	diff	lwr	upr	p adj
**#2-#1**	1.8960873	-2.393499	6.185673	0.4186361
**#3-#1**	2.7736834	-1.515903	7.063270	0.1968933
**#3-#2**	0.8775961	-3.411990	5.167182	0.8109613

Table S5 (continued)

PROM1	Df	Sum Sq	Mean Sq	F value	Pr(>F)
**Medium**	3	1.356	0.4520	0.2551	0.8552
**Individuals**	2	9.240	4.6200	2.6078	0.1531
**Residuals**	6	10.630	1.7716		

multiple comparisons - Medium

PROM1	diff	lwr	upr	p adj
**Diff.-i.c.i.**	0.22296423	-3.539163	3.985092	0.9966093
**Exp.-i.c.i.**	-0.61009966	-4.372227	3.152028	0.9399984
**Ref.-i.c.i.**	0.19762875	-3.564499	3.959756	0.9976269
**Exp.-Diff.**	-0.83306388	-4.595191	2.929064	0.8667215
**Ref.-Diff.**	-0.02533548	-3.787463	3.736792	0.9999949
**Ref.-Exp.**	0.80772841	-2.954399	4.569856	0.8764183

multiple comparisons - Individuals

PROM1	diff	lwr	upr	p adj
**#2-#1**	1.9710925	-0.9167099	4.858895	0.1712435
**#3-#1**	1.7279119	-1.1598905	4.615714	0.2369883
**#3-#2**	-0.2431806	-3.1309831	2.644622	0.9640852

VIL1	Df	Sum Sq	Mean Sq	F value	Pr(>F)	
**Medium**	3	57.900	19.300	56.003	0.00009	***
**Individuals**	2	4.927	2.464	7.149	0.02583	*
**Residuals**	6	2.068	0.345			

multiple comparisons - Medium

VIL1	diff	lwr	upr	p adj
**Diff.-i.c.i.**	-4.9208167	-6.580102	-3.261531	0.0002088
**Exp.-i.c.i.**	-4.9670027	-6.626288	-3.307717	0.0001980
**Ref.-i.c.i.**	-5.2973492	-6.956635	-3.638064	0.0001372
**Exp.-Diff.**	-0.0461861	-1.705471	1.613099	0.9996422
**Ref.-Diff.**	-0.3765325	-2.035818	1.282753	0.8585872
**Ref.-Exp.**	-0.3303465	-1.989632	1.328939	0.8976296

multiple comparisons - Individuals

VIL1	diff	lwr	upr	p adj
**#2-#1**	-0.239679	-1.5133435	1.033986	0.8367428
**#3-#1**	1.223558	-0.0501067	2.497222	0.0580547
**#3-#2**	1.463237	0.1895722	2.736901	0.0288403

Table S6: Statistical analysis of jejunal organoids in different media
Two-way ANOVA, Tukey multiple comparisons of mean, 95 % family-wise confidence level; i.c.i., initial cell isolates; Exp., expansion medium; Diff., differentiation medium; Ref., refined medium; code: 0 *** 0.001 ** 0.01 * 0.05 . 0.1

LGR5	**Df**	**Sum Sq**	**Mean Sq**	**F value**	**Pr(>F)**	
**Medium**	3	36.155	12.0516	2.7531	0.13460	
**Individuals**	2	40.609	20.3044	4.6384	0.06058	.
**Residuals**	6	26.265	4.3775			

multiple comparisons - Medium

LGR5	**diff**	**lwr**	**upr**	**p adj**
**Diff.-i.c.i.**	3.1165489	-2.797123	9.030221	0.3481746
**Exp.-i.c.i.**	4.8419211	-1.071751	10.755593	0.1049447
**Ref.-i.c.i.**	2.5566058	-3.357066	8.470278	0.4935994
**Exp.-Diff.**	1.7253721	-4.188300	7.639044	0.7502072
**Ref.-Diff.**	-0.5599432	-6.473615	5.353729	0.9866617
**Ref.-Exp.**	-2.2853153	-8.198987	3.628357	0.5749125

multiple comparisons - Individuals

LGR5	**diff**	**lwr**	**upr**	**p adj**
**#2-#1**	0.4167766	-4.122548	4.9561009	0.9575005
**#3-#1**	-3.6772251	-8.216549	0.8620992	0.1039745
**#3-#2**	-4.0940017	-8.633326	0.4453226	0.0727493

NEUROG3	**Df**	**Sum Sq**	**Mean Sq**	**F value**	**Pr(>F)**	
**Medium**	3	68.665	22.8882	28.5235	0.0006	***
**Individuals**	2	0.473	0.2363	0.2944	0.7551	
**Residuals**	6	4.815	0.8024			

multiple comparisons - Medium

NEUROG3	**diff**	**lwr**	**upr**	**p adj**
**Diff.-i.c.i.**	1.274379	-1.2575401	3.806298	0.3812656
**Exp.-i.c.i.**	-3.671666	-6.2035854	-1.139747	0.0095567
**Ref.-i.c.i.**	2.796962	0.2650425	5.328881	0.0331531
**Exp.-Diff.**	-4.946045	-7.4779645	-2.414126	0.0020905
**Ref.-Diff.**	1.522583	-1.0093366	4.054502	0.2590550
**Ref.-Exp.**	6.468628	3.9367088	9.000547	0.0004834

multiple comparisons - Individuals

NEUROG3	**diff**	**lwr**	**upr**	**p adj**
**#2-#1**	0.136271	-1.807226	2.079768	0.9749137
**#3-#1**	0.472199	-1.471298	2.415696	0.7472559
**#3-#2**	0.335928	-1.607569	2.279425	0.8597760

Table S6 (continued)

CHGA	Df	Sum Sq	Mean Sq	F value	Pr(>F)	
**Medium**	3	138.637	46.212	21.7246	0.00127	**
**Individuals**	2	20.991	10.495	4.9339	0.05406	.
**Residuals**	6	12.763	2.127			

multiple comparisons - Medium

CHGA	diff	lwr	upr	p adj
**Diff.-i.c.i.**	-6.655476	-10.7778636	-2.533088	0.0056164
**Exp.-i.c.i.**	-8.877927	-13.0003151	-4.755540	0.0012382
**Ref.-i.c.i.**	-3.008825	-7.1312128	1.113562	0.1521652
**Exp.-Diff.**	-2.222451	-6.3448391	1.899936	0.3321387
**Ref.-Diff.**	3.646651	-0.4757368	7.769038	0.0798861
**Ref.-Exp.**	5.869102	1.7467147	9.991490	0.0104465

multiple comparisons - Individuals

CHGA	diff	lwr	upr	p adj
**#2-#1**	3.0287061	-0.1356314	6.193044	0.0588404
**#3-#1**	0.5185096	-2.6458279	3.682847	0.8727269
**#3-#2**	-2.5101965	-5.6745340	0.654141	0.1110407

MUC2	Df	Sum Sq	Mean Sq	F value	Pr(>F)	
**Medium**	3	184.246	61.415	13.9443	0.00411	**
**Individuals**	2	8.035	4.018	0.9122	0.45092	
**Residuals**	6	26.426	4.404			

multiple comparisons - Medium

MUC2	diff	lwr	upr	p adj
**Diff.-i.c.i.**	-4.870446	-10.802236	1.0613433	0.1039407
**Exp.-i.c.i.**	-10.413091	-16.344880	-4.4813015	0.0036616
**Ref.-i.c.i.**	-2.012563	-7.944353	3.9192263	0.6624502
**Exp.-Diff.**	-5.542645	-11.474434	0.3891446	0.0651459
**Ref.-Diff.**	2.857883	-3.073906	8.7896724	0.4133198
**Ref.-Exp.**	8.400528	2.468738	14.3323172	0.0107167

multiple comparisons - Individuals

MUC2	diff	lwr	upr	p adj
**#2-#1**	1.9815210	-2.571710	6.534752	0.4284646
**#3-#1**	1.2523907	-3.300840	5.805622	0.6918295
**#3-#2**	-0.7291303	-5.282361	3.824101	0.8779664

Table S6 (continued)

ASCL2	Df	Sum Sq	Mean Sq	F value	Pr(>F)
**Medium**	3	8.4816	2.8272	1.5257	0.3014
**Individuals**	2	2.9731	1.4865	0.8022	0.4912
**Residuals**	6	11.1187	1.8531		

multiple comparisons - Medium

ASCL2	diff	lwr	upr	p adj
**Diff.-i.c.i.**	2.2007884	-1.646863	6.048440	0.2916274
**Exp.-i.c.i.**	1.8200768	-2.027575	5.667728	0.4268733
**Ref.-i.c.i.**	1.6235295	-2.224122	5.471181	0.5114791
**Exp.-Diff.**	-0.3807116	-4.228363	3.466940	0.9848609
**Ref.-Diff.**	-0.5772589	-4.424910	3.270393	0.9513527
**Ref.-Exp.**	-0.1965473	-4.044199	3.651104	0.9978157

multiple comparisons - Individuals

ASCL2	diff	lwr	upr	p adj
**#2-#1**	-1.1103136	-4.063764	1.843137	0.5196990
**#3-#1**	-0.9913935	-3.944844	1.962057	0.5867853
**#3-#2**	0.1189201	-2.834531	3.072371	0.9916324

EPHB2	Df	Sum Sq	Mean Sq	F value	Pr(>F)
**Medium**	3	8.6974	2.8991	1.3829	0.3356
**Individuals**	2	12.5919	6.2959	3.0032	0.1248
**Residuals**	6	12.5786	2.0964		

multiple comparisons - Medium

EPHB2	diff	lwr	upr	p adj
**Diff.-i.c.i.**	1.6606551	-2.431824	5.753134	0.5399845
**Exp.-i.c.i.**	2.2599590	-1.832520	6.352438	0.3154603
**Ref.-i.c.i.**	1.7654026	-2.327076	5.857881	0.4952134
**Exp.-Diff.**	0.5993038	-3.493175	4.691783	0.9544571
**Ref.-Diff.**	0.1047474	-3.987731	4.197226	0.9997216
**Ref.-Exp.**	-0.4945564	-4.587035	3.597922	0.9732676

multiple comparisons - Individuals

EPHB2	diff	lwr	upr	p adj
**#2-#1**	0.6818513	-2.459528	3.8232307	0.7907144
**#3-#1**	-1.7503088	-4.891688	1.3910707	0.2768419
**#3-#2**	-2.4321600	-5.573540	0.7092195	0.1196388

Table S6 (continued)

BMI1	Df	Sum Sq	Mean Sq	F value	Pr(>F)
**Medium**	3	1.52044	0.50681	1.4246	0.3251
**Individuals**	2	0.66817	0.33408	0.9391	0.4418
**Residuals**	6	2.13452	0.35575		

multiple comparisons - Medium

BMI1	diff	lwr	upr	p adj
**Diff.-i.c.i.**	-0.81817253	-2.504026	0.8676814	0.4079465
**Exp.-i.c.i.**	-0.50543760	-2.191292	1.1804163	0.7356101
**Ref.-i.c.i.**	-0.91155115	-2.597405	0.7743028	0.3300799
**Exp.-Diff.**	0.31273493	-1.373119	1.9985889	0.9145461
**Ref.-Diff.**	-0.09337862	-1.779233	1.5924753	0.9972238
**Ref.-Exp.**	-0.40611355	-2.091967	1.2797404	0.8370240

multiple comparisons - Individuals

BMI1	diff	lwr	upr	p adj
**#2-#1**	-0.5619701	-1.856029	0.7320884	0.4298008
**#3-#1**	-0.1639206	-1.457979	1.1301379	0.9211661
**#3-#2**	0.3980495	-0.896009	1.6921080	0.6351379

NOTCH1	Df	Sum Sq	Mean Sq	F value	Pr(>F)
**Medium**	3	1.7409	0.5803	0.1191	0.9455
**Individuals**	2	0.1425	0.0712	0.0146	0.9855
**Residuals**	6	29.2271	4.8712		

multiple comparisons - Medium

NOTCH1	diff	lwr	upr	p adj
**Diff.-i.c.i.**	0.93646706	-5.301773	7.174707	0.9512749
**Exp.-i.c.i.**	0.92653179	-5.311708	7.164772	0.9526681
**Ref.-i.c.i.**	0.57200384	-5.666236	6.810244	0.9878426
**Exp.-Diff.**	-0.00993527	-6.248176	6.228305	0.9999999
**Ref.-Diff.**	-0.36446322	-6.602703	5.873777	0.9967495
**Ref.-Exp.**	-0.35452795	-6.592768	5.883712	0.9970045

multiple comparisons - Individuals

NOTCH1	diff	lwr	upr	p adj
**#2-#1**	-0.26272963	-5.051192	4.525733	0.9845374
**#3-#1**	-0.17210072	-4.960563	4.616362	0.9933255
**#3-#2**	0.09062891	-4.697833	4.879091	0.9981431

Table S6 (continued)

PROM1	Df	Sum Sq	Mean Sq	F value	Pr(>F)
**Medium**	3	0.1982	0.06605	0.0701	0.9738
**Individuals**	2	0.6922	0.34611	0.3674	0.7071
**Residuals**	6	5.6530	0.94217		

multiple comparisons - Medium

PROM1	diff	lwr	upr	p adj
**Diff.-i.c.i.**	0.10788196	-2.635652	2.851415	0.9989965
**Exp.-i.c.i.**	-0.20140074	-2.944934	2.542133	0.9936361
**Ref.-i.c.i.**	-0.18069289	-2.924226	2.562841	0.9953720
**Exp.-Diff.**	-0.30928270	-3.052816	2.434251	0.9780317
**Ref.-Diff.**	-0.28857485	-3.032108	2.454959	0.9819640
**Ref.-Exp.**	0.02070785	-2.722826	2.764241	0.9999928

multiple comparisons - Individuals

PROM1	diff	lwr	upr	p adj
**#2-#1**	0.5860641	-1.519867	2.691996	0.6861910
**#3-#1**	0.2485591	-1.857372	2.354491	0.9310686
**#3-#2**	-0.3375050	-2.443437	1.768426	0.8777859

VIL1	Df	Sum Sq	Mean Sq	F value	Pr(>F)	
**Medium**	3	22.2150	7.4050	18.8676	0.001855	**
**Individuals**	2	0.8182	0.4091	1.0424	0.408746	
**Residuals**	6	2.3548	0.3925			

multiple comparisons - Medium

VIL1	diff	lwr	upr	p adj
**Diff.-i.c.i.**	3.1139296	-4.884650	-1.343210	0.0036284
**Exp.-i.c.i.**	-2.9015377	-4.672258	-1.130818	0.0052102
**Ref.-i.c.i.**	-3.3475266	-5.118247	-1.576807	0.0024865
**Exp.-Diff.**	0.2123919	-1.558328	1.983112	0.9738231
**Ref.-Diff.**	-0.2335970	-2.004317	1.537123	0.9658403
**Ref.-Exp.**	-0.4459889	-2.216709	1.324731	0.8192909

multiple comparisons - Individuals

VIL1	diff	lwr	upr	p adj
**#2-#1**	-0.312494	-1.6716959	1.046707	0.7693514
**#3-#1**	0.327062	-1.0321392	1.686264	0.7512342
**#3-#2**	0.639557	-0.7196448	1.998758	0.3792827

Table S7: Statistical analysis of colonic organoids in different media

Two-way ANOVA, Tukey multiple comparisons of mean, 95 % family-wise confidence level; i.c.i., initial cell isolates; Exp., expansion medium; Diff., differentiation medium; Ref., refined medium; code: 0 *** 0.001 ** 0.01 * 0.05 . 0.1

LGR5	**Df**	**Sum Sq**	**Mean Sq**	**F value**	**Pr(>F)**
**Medium**	3	26.2825	8.7608	3.1277	0.1090
**Individuals**	2	9.5059	4.7529	1.6968	0.2606
**Residuals**	6	16.8062	2.8010		

multiple comparisons - Medium

LGR5	**diff**	**lwr**	**upr**	**p adj**
**Diff.-i.c.i.**	0.2086861	-4.5217908	4.939163	0.9985868
**Exp.-i.c.i.**	-2.8736011	-7.6040780	1.856876	0.2526962
**Ref.-i.c.i.**	1.0531542	-3.6773227	5.783631	0.8649645
**Exp.-Diff.**	-3.0822872	-7.8127640	1.648190	0.2108279
**Ref.-Diff.**	0.8444681	-3.8860088	5.574945	0.9226583
**Ref.-Exp.**	3.9267553	-0.8037216	8.657232	0.1001101

multiple comparisons - Individuals

LGR5	**diff**	**lwr**	**upr**	**p adj**
**#2-#1**	1.758482	-1.872624	5.389588	0.3613051
**#3-#1**	1.995264	-1.635842	5.626370	0.2848942
**#3-#2**	0.236782	-3.394324	3.867888	0.9782524

NEUROG3	**Df**	**Sum Sq**	**Mean Sq**	**F value**	**Pr(>F)**	
**Medium**	3	116.046	38.682	9.0616	0.01202	*
**Individuals**	2	7.246	3.623	0.8487	0.47362	
**Residuals**	6	25.612	4.269			

multiple comparisons - Medium

NEUROG3	**diff**	**lwr**	**upr**	**p adj**
**Diff.-i.c.i.**	2.4722754	-3.367491	8.3120415	0.5090485
**Exp.-i.c.i.**	-5.2677304	-11.107497	0.5720357	0.0743561
**Ref.-i.c.i.**	2.2147303	-3.625036	8.0544964	0.5881030
**Exp.-Diff.**	-7.7400058	-13.579772	-1.9002397	0.0146759
**Ref.-Diff.**	-0.2575451	-6.097311	5.5822210	0.9985881
**Ref.-Exp.**	7.4824607	1.642695	13.3222268	0.0171722

multiple comparisons - Individuals

NEUROG3	**diff**	**lwr**	**upr**	**p adj**
**#2-#1**	-0.1272758	-4.609870	4.355318	0.9958273
**#3-#1**	1.5810301	-2.901564	6.063624	0.5580088
**#3-#2**	1.7083059	-2.774288	6.190900	0.5113629

Table S7 (continued)

CHGA	Df	Sum Sq	Mean Sq	F value	Pr(>F)	
**Medium**	3	140.348	46.783	16.8359	0.002512	**
**Individuals**	2	4.306	2.153	0.7749	0.501940	
**Residuals**	6	16.673	2.779			

multiple comparisons - Medium

CHGA	diff	lwr	upr	p adj
**Diff.-i.c.i.**	-3.168514	-7.880132	1.543104	0.1933241
**Exp.-i.c.i.**	-8.899055	-13.610672	-4.187437	0.0024986
**Ref.-i.c.i.**	-1.172461	-5.884079	3.539157	0.8242425
**Exp.-Diff.**	-5.730540	-10.442158	-1.018922	0.0217627
**Ref.-Diff.**	1.996054	-2.715564	6.707672	0.5085394
**Ref.-Exp.**	7.726594	3.014976 1	2.438212	0.0051896

multiple comparisons - Individuals

CHGA	diff	lwr	upr	p adj
**#2-#1**	1.139112	-2.477518	4.755742	0.6223666
**#3-#1**	1.370639	-2.245991	4.987269	0.5147721
**#3-#2**	0.231527	-3.385103	3.848157	0.9790290

MUC2	Df	Sum Sq	Mean Sq	F value	Pr(>F)	
**Medium**	3	294.954	98.318	16.3704	0.002705	**
**Individuals**	2	69.844	34.922	5.8146	0.039423	*
**Residuals**	6	36.035	6.006			

multiple comparisons - Medium

MUC2	diff	lwr	upr	p adj
**Diff.-i.c.i.**	-7.727649	-14.654442	-0.8008552	0.0317877
**Exp.-i.c.i.**	-13.586190	-20.512984	-6.6593969	0.0020460
**Ref.-i.c.i.**	-4.410550	-11.337343	2.5162440	0.2241440
**Exp.-Diff.**	-5.858542	-12.785335	1.0682519	0.0937952
**Ref.-Diff.**	3.317099	-3.609694	10.2438928	0.4177941
**Ref.-Exp.**	9.175641	2.248847	16.1024344	0.0147141

multiple comparisons - Individuals

MUC2	diff	lwr	upr	p adj
**#2-#1**	5.896619	0.5796244	11.213614	0.0333325
**#3-#1**	2.610940	-2.7060548	7.927934	0.3526861
**#3-#2**	-3.285679	-8.6026738	2.031315	0.2198710

Table S7 (continued)

ASCL2	Df	Sum Sq	Mean Sq	F value	Pr(>F)	
**Medium**	3	39.682	13.2272	5.7120	0.03423	*
**Individuals**	2	0.774	0.3871	0.1672	0.84986	
**Residuals**	6	13.894	2.3157			

multiple comparisons - Medium

ASCL2	diff	lwr	upr	p adj
**Diff.-i.c.i.**	4.5324837	0.2313096	8.833658	0.0404105
**Exp.-i.c.i.**	1.4989514	-2.8022227	5.800126	0.6452220
**Ref.-i.c.i.**	3.8590898	-0.4420843	8.160264	0.0758444
**Exp.-Diff.**	-3.0335323	-7.3347064	1.267642	0.1686391
**Ref.-Diff.**	-0.6733939	-4.9745681	3.627780	0.9454069
**Ref.-Exp.**	2.3601384	-1.9410358	6.661312	0.3198500

multiple comparisons - Individuals

ASCL2	diff	lwr	upr	p adj
**#2-#1**	0.52648460	-2.775089	3.828058	0.8789002
**#3-#1**	0.55041363	-2.751160	3.851987	0.8686637
**#3-#2**	0.02392903	-3.277645	3.325503	0.9997274

EPHB2	Df	Sum Sq	Mean Sq	F value	Pr(>F)	
**Medium**	3	6.4322	2.14406	5.2372	0.04108	*
**Individuals**	2	0.1506	0.07530	0.1839	0.83652	
**Residuals**	6	2.4564	0.40939			

multiple comparisons - Medium

EPHB2	diff	lwr	upr	p adj
**Diff.-i.c.i.**	0.3301723	-1.47831593	2.1386605	0.9180054
**Exp.-i.c.i.**	-1.3531360	-3.16162424	0.4553522	0.1409258
**Ref.-i.c.i.**	0.5176058	-1.29088246	2.3260940	0.7601687
**Exp.-Diff.**	-1.6833083	-3.49179654	0.1251799	0.0661121
**Ref.-Diff.**	0.1874335	-1.62105476	1.9959217	0.9827098
**Ref.-Exp.**	1.8707418	0.06225355	3.6792300	0.0436101

multiple comparisons - Individuals

EPHB2	diff	lwr	upr	p adj
**#2-#1**	0.07108868	-1.317104	1.459281	0.9865122
**#3-#1**	0.26506998	-1.123122	1.653262	0.8324739
**#3-#2**	0.19398130	-1.194211	1.582174	0.9051761

Table S7 (continued)

BMI1	Df	Sum Sq	Mean Sq	F value	Pr(>F)	
**Medium**	3	1.62206	0.54069	5.1498	0.04254	*
**Individuals**	2	0.50506	0.25253	2.4052	0.17097	
**Residuals**	6	0.62995	0.10499			

multiple comparisons - Medium

BMI1	diff	lwr	upr	p adj
**Diff.-i.c.i.**	-0.4571877	-1.3730342	0.45865887	0.3872676
**Exp.-i.c.i.**	-1.0014269	-1.9172735	-0.0855804	0.0346232
**Ref.-i.c.i.**	-0.7112813	-1.6271279	0.20456519	0.1251251
**Exp.-Diff.**	-0.5442393	-1.4600858	0.37160725	0.2666303
**Ref.-Diff.**	-0.2540937	-1.1699402	0.66175285	0.7757058
**Ref.-Exp.**	0.2901456	-0.6257009	1.20599213	0.7043471

multiple comparisons - Individuals

BMI1	diff	lwr	upr	p adj
**#2-#1**	0.2005821	-0.5024201	0.9035843	0.6739345
**#3-#1**	-0.2987368	-1.0017390	0.4042654	0.4434886
**#3-#2**	-0.4993189	-1.2023211	0.2036833	0.1536914

NOTCH1	Df	Sum Sq	Mean Sq	F value	Pr(>F)
**Medium**	3	5.9351	1.97835	3.2214	0.1037
**Individuals**	2	0.2126	0.10631	0.1731	0.8451
**Residuals**	6	3.6848	0.61413		

multiple comparisons - Medium

NOTCH1	diff	lwr	upr	p adj
**Diff.-i.c.i.**	1.4992568	-0.7157564	3.7142699	0.1898429
**Exp.-i.c.i.**	-0.3131459	-2.5281591	1.9018672	0.9586432
**Ref.-i.c.i.**	0.7631995	-1.4518136	2.9782126	0.6525893
**Exp.-Diff.**	-1.8124027	-4.0274158	0.4026104	0.1051774
**Ref.-Diff.**	-0.7360573	-2.9510704	1.4789559	0.6754961
**Ref.-Exp.**	1.0763454	-1.1386677	3.2913586	0.4070133

multiple comparisons - Individuals

NOTCH1	diff	lwr	upr	p adj
**#2-#1**	0.1904815	-1.509759	1.890722	0.9376041
**#3-#1**	-0.1339348	-1.834175	1.566305	0.9684763
**#3-#2**	-0.3244164	-2.024657	1.375824	0.8326907

Table S7 (continued)

PROM1	Df	Sum Sq	Mean Sq	F value	Pr(>F)	
**Medium**	3	6.0498	2.01661	3.6915	0.08137	.
**Individuals**	2	4.8039	2.40193	4.3969	0.06671	.
**Residuals**	6	3.2777	0.54628			

multiple comparisons - Medium

PROM1	diff	lwr	upr	p adj
**Diff.-i.c.i.**	-1.3063555	-3.395425	0.7827138	0.2349027
**Exp.-i.c.i.**	-1.9158485	-4.004918	0.1732208	0.699143
**Ref.-i.c.i.**	-1.4400190	-3.529088	0.6490503	0.1802548
**Exp.-Diff.**	-0.6094929	-2.698562	1.4795763	0.7502190
**Ref.-Diff.**	-0.1336635	-2.222733	1.9554058	0.9957500
**Ref.-Exp.**	0.4758294	-1.613240	2.5648987	0.8573153

multiple comparisons - Individuals

PROM1	diff	lwr	upr	p adj
**#2-#1**	0.7097294	-0.8938366	2.31329529	0.4178180
**#3-#1**	-0.8383094	-2.4418753	0.76525655	0.3144299
**#3-#2**	-1.5480387	-3.1516047	0.05552718	0.0570207

VIL1	Df	Sum Sq	Mean Sq	F value	Pr(>F)	
**Medium**	3	16.9034	5.6345	12.2278	0.005746	**
**Individuals**	2	3.5977	1.7989	3.9039	0.082051	
**Residuals**	6	2.7647	0.4608			

multiple comparisons - Medium

VIL1	diff	lwr	upr	p adj
**Diff.-i.c.i.**	-2.4805094	-4.399165	-0.561854	0.0164759
**Exp.-i.c.i.**	-2.7677175	-4.686373	-0.849062	0.0098042
**Ref.-i.c.i.**	-2.9055920	-4.824248	-0.986936	0.0077294
**Exp.-Diff.**	-0.2872080	-2.205864	1.631448	0.9516488
**Ref.-Diff.**	-0.4250826	-2.343738	1.493573	0.8665487
**Ref.-Exp.**	-0.1378745	-2.056530	1.780781	0.9940217

multiple comparisons - Individuals

VIL1	diff	lwr	upr	p adj
**#2-#1**	0.243242	-1.229514	1.715999	0.8708792
**#3-#1**	-1.020649	-2.493405	0.452108	0.1644002
**#3-#2**	-1.263891	-2.736648	0.208866	0.0861859
